# Proteoglycan-targeting applied to hypoxia-activated prodrug therapy in chondrosarcoma: first proof-of-concept

**DOI:** 10.18632/oncotarget.21337

**Published:** 2017-09-27

**Authors:** Aurélien Voissiere, Valérie Weber, Yvain Gerard, Françoise Rédini, Florian Raes, Jean-Michel Chezal, Françoise Degoul, Caroline Peyrode, Elisabeth Miot-Noirault

**Affiliations:** ^1^ Université Clermont Auvergne, INSERM, U1240 Imagerie Moléculaire et Stratégies Théranostiques, Clermont-Ferrand, France; ^2^ Nantes Atlantique Université, INSERM, UMR1238, Sarcomes osseux et remodelage des tissus calcifiés, Nantes, France; ^3^ PHENOMIN-TAAM-UPS44, CIPA (Centre d’Imagerie du Petit Animal), CNRS Orléans, France

**Keywords:** chondrosarcoma, proteoglycan, hypoxia-activated prodrug, quaternary ammonium, extracellular matrix

## Abstract

Due to its abundant chondrogenic matrix and hypoxic tissue, chondrosarcoma is chemo- and radio-resistant. Our group has developed a proteoglycan targeting strategy by using a quaternary ammonium (QA) function as a carrier of DNA alkylating agents to chondrosarcoma environment. Here, we assessed the relevance of this strategy applied to hypoxia-activated prodrugs, by conjugating a QA to 2-nitroimidazole phosphoramidate. This derivative, named as **8-QA**, was evaluated respectively to its non-QA equivalent and to a QA-conjugated but non-hypoxia activated. Firstly binding to aggrecan was confirmed from dissociation constant determined by Surface Plasmon Resonance. *In vitro*, in HEMC-SS chondrosarcoma cells cultured in monolayer and in spheroids, **8-QA** showed higher cytotoxic activity in hypoxia versus normoxia, and led to a strong accumulation of cells in S phase and apoptosis. *In vivo*, a HEMC-SS xenograft model was implanted on SCID mice and characterized for hypoxia by photoacoustic imaging as well as proteoglycan content. When HEMC-SS bearing mice were given **8-QA** at 47 μmol/kg according to a q4d x 6 schedule, a significant 62.1% inhibition of tumor growth was observed, without associated hematological side effects. Mechanistic studies of treated tumors highlighted decrease in mitotic index associated to increase in both p21 and p53S15 markers. Interestingly, **8-QA** treatment induced an increase of DNA damages as measured by γH2AX predominantly found in pimonidazole-positive hypoxic areas. These preclinical results are the first to demonstrate the interest of addressing hypoxia-activated prodrugs selectively to proteoglycan of chondrogenic tumor tissue, as a promising therapeutic strategy.

## INTRODUCTION

Chondrosarcoma is a malignant cartilage-forming tumor that is the second most common type of skeletal malignancy after osteosarcoma, but the first in adults [1]. As chemo- or radio-therapies are ineffective due to the tumor's extracellular matrix (ECM), low percentage of dividing cells and poor vascularity, surgery with wide margins remains the mainstay of treatment.

Given the poor patient outcomes (10-year survival rate of 29%–83% depending of grading), personalized medicine including a combination of targeted therapies has been suggested as an adjuvant to surgical excision for inoperable primary or recurrent diseases [2, 3].

There is a growing body of evidence that targeting the tumor microenvironment could complement traditional conventional treatments and improve therapeutic outcomes [4, 5]. Apart from the tumor cells, the tumor microenvironment including surrounding blood vessels, ECM, other non-malignant cells and signaling molecules largely contribute to almost all physical and chemical hallmarks of cancer [6, 7].

For many years, the hypoxic tumor microenvironment has attracted intensive research as a potential target for anti-cancer therapies, with two main approaches being developed:

(i) molecular-targeted drugs designed to exploit biochemical responses to hypoxia, particularly hypoxia-inducible factor pathways [8];

(ii) “bioreductive drugs” or “hypoxia-activated prodrugs” (HAP). The modular concept of HAP design involves three main components—a trigger, a linker, and an effector. The effector is the cytotoxic component capable of killing cells within the hypoxic microenvironment and predominately belongs to the class of potent DNA-alkylating agents. The purpose of the linker is to deactivate the effector. The trigger group is the critical group that determines prodrug activation and hypoxia selectivity. Under low-oxygen conditions, the effector is selectively released after reduction [9]. The five classes commonly described for HAP count a number of nitro (hetero) aromatic compounds, such as the 2-nitroimidazole HAP of bromo-isophosphoramide, TH-302, which is currently considered as the gold standard. This HAP is in clinical trials, with phase-I studies published from 2011 and phase III ongoing against soft tissue sarcoma (NCT01440088 study) and pancreatic cancer (NCT01746979, MAESTRO study) [10, 11].

Drugs targeting the hypoxic microenvironment can only be effective if they physically reach their targets. Exploiting the physical and chemical properties of the extracellular matrix of chondrosarcoma, composed of fibrous structural and adhesive proteins as well as proteoglycans, may be a way to complement these HAP therapies. Our team working on ECM-targeting approaches has patented the use of a quaternary ammonium (QA) function as Proteoglycan-targeting ligand [12]. Due to the high number of sulfate and carboxylate groups of their glycosaminoglycan moieties, chondrosarcoma proteoglycans have strong negative charges that may interact with the positively-charged QA function. We have previously demonstrated the proof of concept that QA could act as a carrier to deliver agents to chondrosarcoma, such as radiopharmaceuticals for imaging or DNA-alkylating agents for therapy [13–17]. We hypothesized that QA ligands could be conjugated to HAP in order to propose an innovative concept of dual targeting therapy for chondrosarcoma [18–20].

In this study, a QA conjugate of phosphoramidate bearing a nitroimidazole as hypoxia-sensitive function (named **8-QA**) was synthesized and evaluated against its non-QA equivalent (named **8**) and a non-hypoxia activated QA derivative (**10-QA**) on the basis of: (i) affinity for aggrecan determined by surface plasmon resonance (SPR); (ii) cytotoxic activity under normoxic and hypoxic conditions on human HEMC-SS chondrosarcoma cells cultured in monolayer and in spheroids. The mechanism of action of **8-QA** was also determined on the basis of cell cycle arrest and apoptosis study. We then determined *in vivo* efficacy on the HEMC-SS xenograft model implanted on Severe Combined ImmunoDeficiency (SCID) mice by tumor volume monitoring, anatomopathology, western blot and immunohistochemistry for hypoxia marker (pimonidazole) and γH2AX marker of DNA double strand break. Adverse side effects were characterized by mouse weight records, hematological analyses, and histological study of articular cartilage.

## RESULTS

### QA-derivatives bind to aggrecan

**Figure 1:**
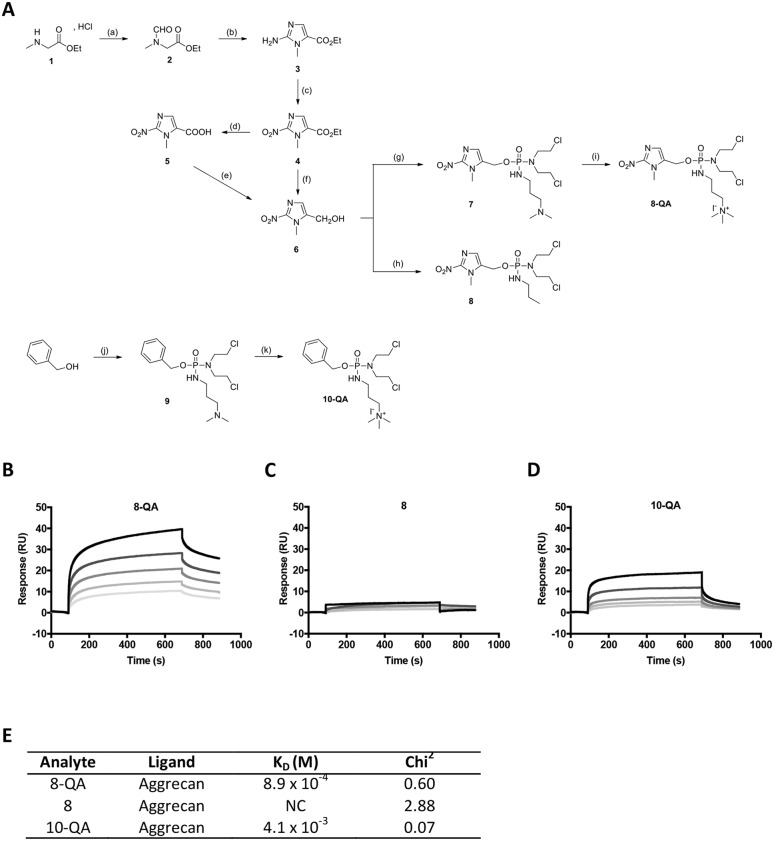
HAP synthesis and binding to immobilized aggrecan by SPR **(A)** Scheme of HAP synthesis. Reagents and conditions : (a) ethylformate, K_2_CO_3_, EtOH, rt, 15-20 hours, **92%**; (b) (1) ethylformate, NaH, 0 °C, rt, 15-20 hours, (2) HCl 37%, EtOH, 110 °C, 1 hour, (3) NH_2_CN, AcONa/AcOH, 95°C, 1 hour, **55%**; (c) NaNO_2_, AcOH, rt, 15-20 hours, **75%**; (d) NaOH, rt, 12 hours, **77%**; (e) (1) *iso*butylchloroformate, TEA, THF anh., -20°C, 2 hours, (2) NaBH_4_, H_2_O, -20 to -10°C, 1.5 hours, **75%**; (f) NaBH_4_, LiBr, THF/MeOH/H_2_O, 0°C, rt, 7 hours, **70%**; (g) (1) bis(2-chloroethyl)phosphoramidic dichloride, Li(TMS)_2_N, THF anh., -78°C, 1 hour, (2) *N,N*-dimethylamino-1-propylamine, THF anh., -78°C, 1 hour, **48%**; (h) (1) bis(2-chloroethyl)phosphoramidic dichloride, Li(TMS)_2_N, THF anh., -78°C, 1.7 hours, (2) *n*-propylamine, THF anh., -78°C, 20 minutes, **65%**; (i) MeI, THF anh., 3.5 hours, **99%**; (j) (1) NaH, bis(2-chloroethyl)phosphoramidic dichloride, toluene anh., 0°C, 15-20 hours, (2) *N,N*-dimethylamino-1-propylamine, 0°C, 2 hours, **88%**; (k) MeI, THF anh., 3.5 hours, **78%**. **(B-E) SPR assays:** Representative sensorgrams of **8-QA** (B), **8** (C) and **10-QA** (D) binding to aggrecan. All derivatives were injected at 1, 0.5, 0.25, 0.125, 0.0625 mM (from top to bottom) during 600 seconds on immobilized aggrecan at a flow rate of 30 μL/min. E. Dissociation constants (K_D_) were determined for each derivative by “steady-state affinity analysis”. All experiments were carried out in triplicate. NC: Non-calculable

The interaction between QA-conjugated derivatives and aggrecan was validated by SPR analyses on a Biacore T200 biosensor. The sensorgrams (Figures 1B to 1D) for HAP derivatives were obtained by subtracting the responses measured in the aggrecan immobilized flow cell with the response measured on the negative control flow cell (without immobilized aggrecan) for potential non-specific binding. After injections of increasing concentrations of analytes, the results showed a dose-dependent binding of QA derivatives **8-QA** and **10-QA** to aggrecan (Figure 1B and 1D) compared to the non-QA conjugated **8** (Figure 1C). Dissociation constants (K_D_) are given in Figure 1E and are 8.9 × 10^-4^ M for **8-QA** and 4.1 × 10^-3^ M for **10-QA.** These results validate the interest of the QA entity for aggrecan targeting.

.

### HEMC-SS spheroids exhibit proteoglycan-rich ECM and a hypoxic environment

**Figure 2:**
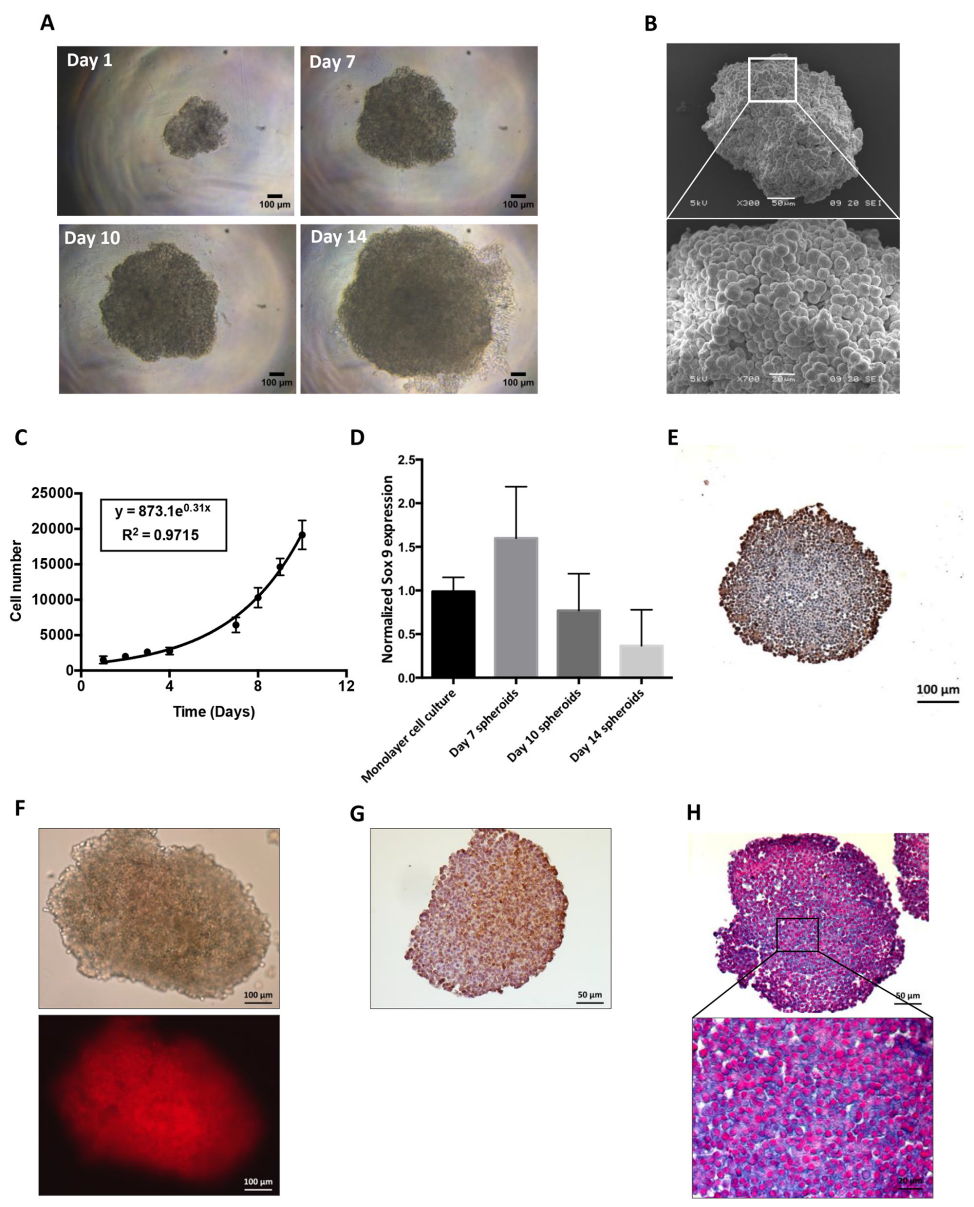
Characterization of HEMC-SS spheroids in terms of growth, hypoxia and proteoglycans **(A)** Optical visualization of HEMC-SS spheroids at different stages of growth (Day 1, 7, 10 and 14). **(B)** SEM visualization of HEMC-SS spheroid at Day 10. **(C)** Growth–time curve of spheroids. Cell number was determined after spheroid trypsinization. **(D)** SOX-9 expression determined by qRT-PCR on HEMC-SS monolayer cell culture and on spheroids at Day 7, Day 10 and and Day 14. Data are represented as mean ± SD. **(E)** Ki-67 immunostaining on Day 10 spheroid section. Proliferative cells were mainly localized at the periphery of the spheroid. **(F)** Representative images of hypoxia on Day 10 spheroid using a LOX-1 probe for which fluorescent signal is only activated by hypoxic conditions. The LOX-1 hypoxia probe was added to spheroid culture medium at a final concentration of 200 μM, 24 hours before fluorescence detection. **(G)** Representative image of pimonidazole immunostaining of hypoxia on Day 10 spheroid section. Pimonidazole was added to spheroid at a final concentration of 200 μM one hour before fixation and paraffin-embedding. **(H)** Alcian blue staining on Day 10 HEMC-SS spheroid sections to evidence proteoglycans.

Spheroids reproduced some of the features of solid tumors such as cell–cell and cell–matrix interactions and chemical and biological factor gradient distributions. A HEMC-SS spheroid model was developed to evaluate the cytotoxic activity of HAP. From Day 1 to Day 14, spheroid growth reflecting cell proliferation was clearly visualized by optical microscopy (Figure 2A). Three-dimensional architecture and cell–cell interactions of the HEMC-SS spheroids were confirmed by SEM (Figure 2B). From Day 1 to Day 10, spheroid growth followed an exponential law associated to a doubling time of 2.5 ± 0.4 days (Figure 2C). The expression of the chondrocyte differentiation marker SOX-9 was confirmed in Day 7 spheroids (Figure 2D). This expression decreased with spheroid growth, in a dedifferentiation process that is commonly observed *in vivo*.

Immunostaining analyses performed on Day 10 spheroid section, evidenced Ki-67 positive cells mainly localized in the periphery (Figure 2E). The hypoxic environment of HEMC-SS spheroids was then characterized using both the hypoxia fluorescent probe LOX-1 and pimonidazole immunostaining. As illustrated in Figure 2F, a strong fluorescent signal was evidenced on Day 10 spheroid and further confirmed by intense pimonidazole-positive immunostaining areas mainly localized in the center of the spheroid section (Figure 2G).

In addition, Alcian blue staining highlighted the presence of proteoglycans in spheroids (Figure 2H).

These results validate the HEMC-SS spheroids as an *in vitro* model with hypoxic areas and proteoglycan contents that could be used to test the HAP.

### 8 and 8-QA exhibit a higher cytotoxic activity in hypoxia

**8** and **8-QA** derivatives caused a significant cell growth inhibition in both HEMC-SS culture models, with a higher activity observed in hypoxia compared to normoxia (Table 1). **8-QA** showed a hypoxic cytotoxicity ratio (HCR) of 6.8 for monolayer cultures and 3.1 for spheroid cultures. Moreover, with **8-QA**, the spheroid/monolayer cytotoxicity ratio (S/M) was 3.4 in normoxia and 7.4 in hypoxia. These results also highlighted that 3D spheroid culture induces a resistance to HAP treatments. For the non-QA derivative **8**, HCR was 7.5 for monolayer cultures and 17.4 for spheroid cultures.

**Table 1:** Cytotoxicity of HAP derivatives on HEMC-SS culture models in normoxic (21% O_2_) and hypoxic (N_2_, O_2_ < 0.3%) conditions

	IC_50_ (μM)							
	Monolayer			Spheroids			S/M	
	Normoxia	Hypoxia	HCR	Normoxia	Hypoxia	HCR	Normoxia	Hypoxia
**8-QA**	29.3 ± 8.0	4.3 ± 1.2^*^	**6.8**	101.1 ± 32.6	31.8 ± 5.5^*^	**3.1**	**3.4**	**7.4**
**8**	32.5 ± 4.3	4.3 ± 1.2^*^	**7.5**	99.4 ± 5.2	5.7 ± 1.2^*^	**17.4**	**3.1**	**1.3**
**10-QA**	> 200	> 200	**NC**	> 200	> 200	**NC**	**NC**	**NC**

Cultures were exposed to drugs for 24 hours followed by incubation for 48 hours in normoxia.

Notes: Data are represented as mean ± SD. ^*^significant difference *versus* normoxic condition.

Abbreviations: IC_50_: Inhibiting concentration 50; HCR: Hypoxic–cytotoxic ratio; S/M: spheroid/monolayer cytotoxic ratio; NC: Not calculable

As expected, the non-hypoxia activated derivative **10-QA** showed no cytotoxicity in any of the experimental conditions (IC_50_ > 200 μM).

### Characterization of 8-QA cytotoxic activity on HEMC-SS cells

**Figure 3:**
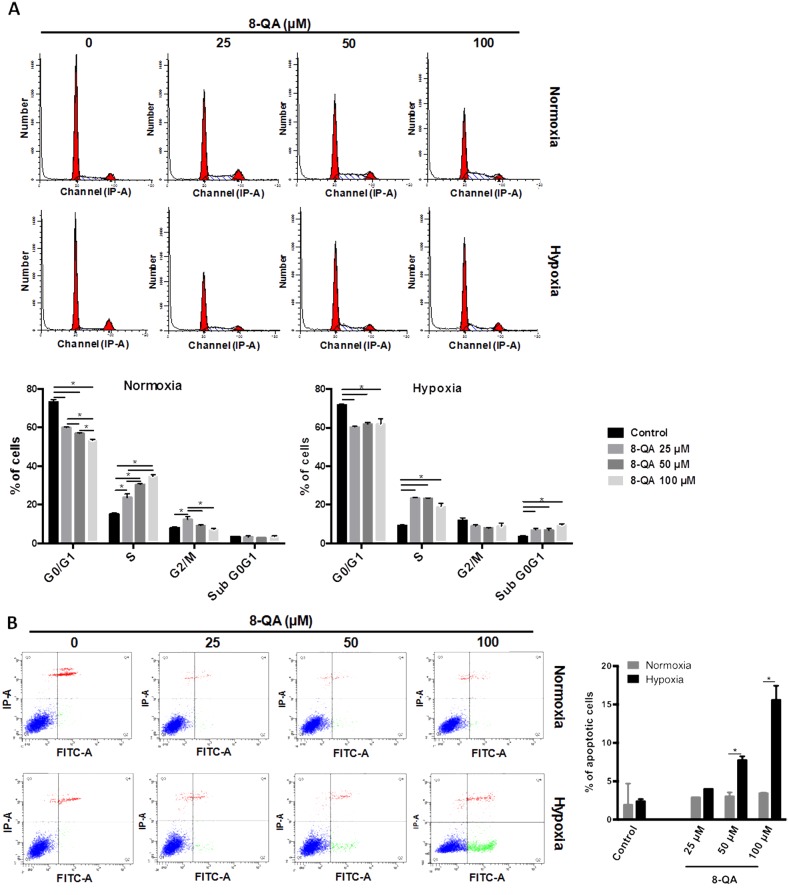
Cell cycle and apoptosis analyses on HEMC-SS cells treated by 8-QA Cell cycle distribution **(A)** and Annexin V test **(B)** on HEMC-SS cells treated in normoxic (21% O_2_) or hypoxic (N_2_, O_2_ < 0.3%) conditions by derivative **8-QA** at 25 μM, 50 μM and 100 μM for 24 hours. (A) In normoxia, **8-QA** treatment led to an increase in S-phase associated with a decrease in G2/M phase. In hypoxia, **8-QA** treatment led to a high accumulation of cells in S-phase associated with an increase in SubG0/G1 phase. Data are represented as mean ± SD. **8-QA** treatments and controls were compared using two-way ANOVA analysis followed by Tukey's post-hoc multiple comparisons test. (B) Increase of Annexin V positive cells was observed after **8-QA** treatment in hypoxia from 50 μM. Data are represented as mean ± SD. Treatments in normoxic and hypoxic conditions were compared using a two-way ANOVA analysis followed by Bonferroni's post-hoc comparisons test.

In order to characterize the mechanism of action of derivative **8-QA**, cell cycle analysis and apoptotic cells were quantified on the human HEMC-SS chondrosarcoma cell line. As illustrated in Figure 3, in normoxia, **8-QA** treatment led to an increase in S-phase (p < 0.001) associated with a decrease in G2/M phase, both in dose-dependent manner (p < 0.05) (Figure 3A). In hypoxia, **8-QA** led to a high accumulation of cells in S and SubG0/G1 phases respectively to controls. An increased in Annexin V positive cells, as the consequence of apoptotic cascade and phosphatidylserine externalization, was observed after **8-QA** treatment in hypoxia from 50 μM (i.e. 4.1-fold cell death at 100 μM, p < 0.001) (Figure 3B).

### Human chondrosarcoma HEMC-SS xenograft model implanted on SCID mice exhibits hypoxia and proteoglycans

**Figure 4:**
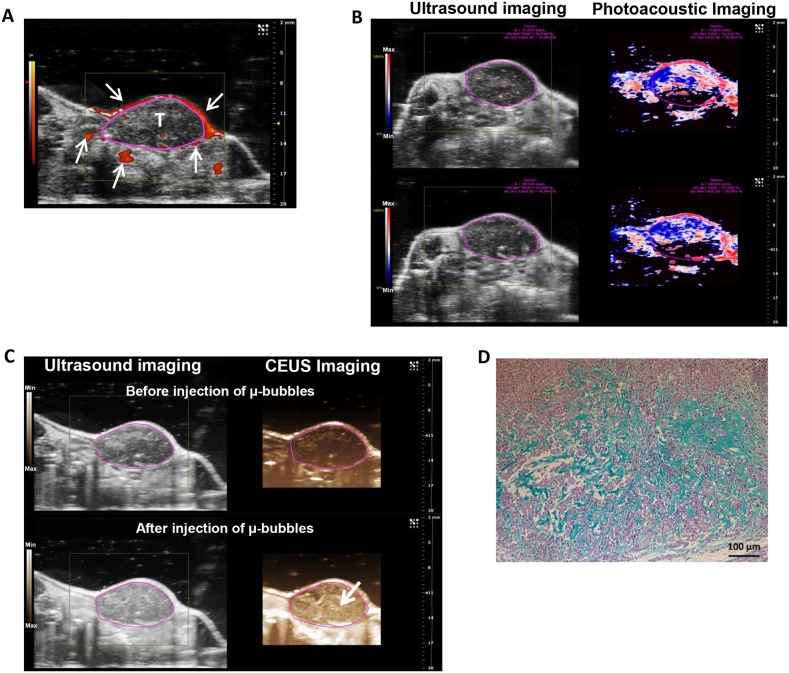
Evaluation and characterization of hypoxia and proteoglycan-rich ECM of HEMC-SS bearing mice **(A)** Representative Doppler imaging for vascularization assessment. Arrows show large vessels mainly localized at the periphery of the tumor (T) delineated by circle. **(B)** Ultrasound (left) and photoacoustic (right) imaging of tumor. On photoacoustic imaging, red color represents high oxygenated areas (max) while blue/black colors represent low oxygenated areas (min). Pink circles delineate the tumor. A global tumor hypoxia fraction of 67 ± 4 % (for a tumor volume of 333 ± 41 mm^3^) was determined using the OxyHemo-Mode. Experiment was performed on 4 independent mice. **(C)** Ultrasound (left) and contrast-enhanced ultrasound (CEUS) (right) imaging before (top) or after (bottom) i.v. injection of μ-bubbles to determine tumor perfusion status. After μ-bubbles injections, higher contrasted pixels (arrow) were observed in the tumor confirming the presence of microvascularization. **(D)** Alcian Blue staining of tumor sections to evidence proteoglycan in ECM.

To evaluate the antitumor efficacy of HAP vectorized to chondrosarcoma proteoglycans, we characterized the human chondrosarcoma HEMC-SS xenograft model implanted on SCID mice in terms of proteoglycan content and hypoxia. First, Doppler imaging evidenced the presence of large vessels preferentially in the tumor periphery (Figure 4A, arrows). Photoacoustic imaging confirmed a highly hypoxic tumor, with a global hypoxic fraction of 67 ± 4% for a tumor volume of 333 ± 41 mm^3^ (Figure 4B). Finally, contrast imaging after i.v. μ-bubble injections highlighted the presence of a microvasculature in the tumors (Figure 4C, lower panel, arrow) confirming that this model is hypoxic and non-necrotic, allowing thus drug distribution throughout in the tumor tissue (Figure 4C).

Finally, Alcian blue staining confirmed the presence of proteoglycans in the ECM of tumor tissue (Figure 4D).

### *In vivo*, the derivative 8-QA induces a significant inhibition of tumor growth

**Figure 5:**
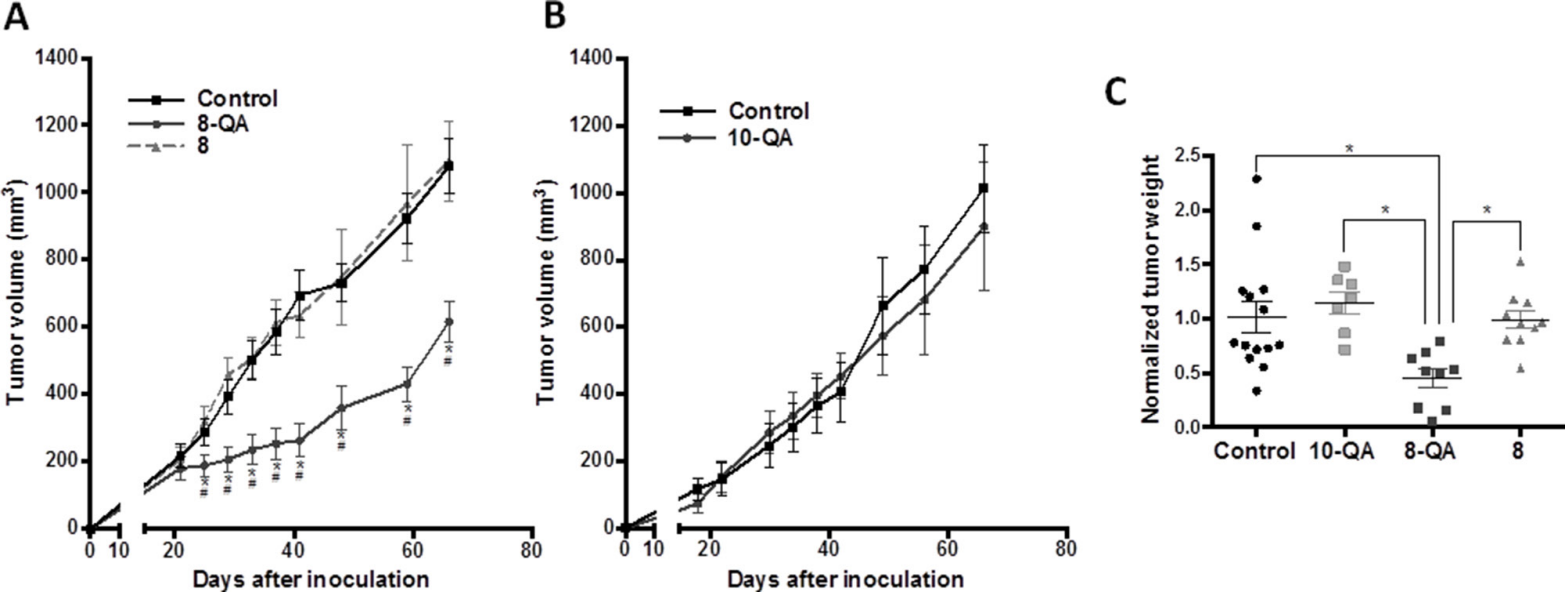
Evaluation of HAP antitumor efficacies on the HEMC-SS xenograft model **(A)** Evaluation of tumor volume after 6 i.v. injections at 4-day intervals of **8-QA** (47.0 μmol/kg) and **8** (47.0 μmol/kg). **(B)** Evaluation of tumor volume after 6 i.v. injections at 4-day intervals of derivative **10-QA** (46.7 μmol/kg). For control groups, vehicle was injected. Data are represented as mean ± SD. ^*^significant difference *versus* control group; ^#^ significant difference *versus* **8**-treated group. **(C)** Weight of tumors sampled one day after the last dose and normalized to control group of each study. Data are represented as mean ± SD.

After validating the hypoxic and proteoglycan-rich microenvironment of the HEMC-SS xenograft model implanted on SCID mice, we first assessed the antitumor efficacy of **8-QA** respectively to non-QA derivative (**8**), used as negative control for proteoglycan targeting**. 8-QA** exhibited a significant antitumor effect from Day 25 to end-of-study with a TGI_D41_ of 62.1% (Figure 5A), while derivative **8** had no antitumor efficacy. In a second independent study, we analyzed the antitumor efficacy of non-hypoxia activated derivative **10-QA** used as negative control for the activation in hypoxic conditions. As expected, this compound had no antitumor effect compared to controls (Figure 5B).

These results were confirmed by tumor weight assessment (Figure 5C), one day after the last injection, with significant decrease of **8-QA** treated tumors as compared to controls (p = 0.008), **8** (p = 0.021) and **10-QA** (p = 0.005).

### Characterization of *in vivo* antitumor efficacy

**Figure 6:**
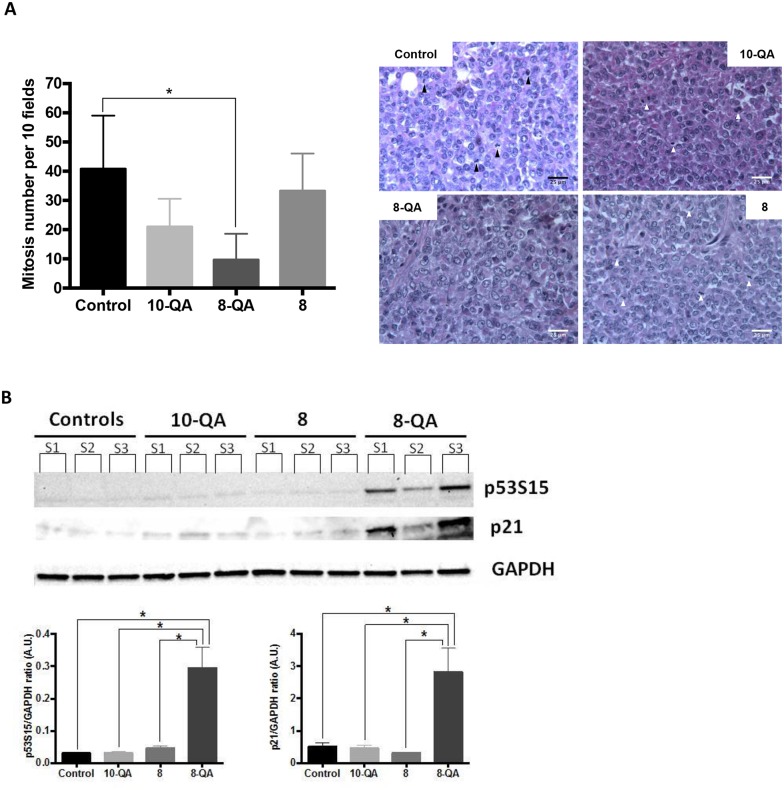
Characterization of *in vivo* antitumor efficacy (**A)** Quantification of mitosis number (left) per 10 fields after H&E staining of paraffin-embedded treated tumor sections (n≥3 tumors per group of treatment). Data are represented as mean ± SD. Representative images of H&E staining (right). Arrows show mitosis. **(B)** Western blot analyses of the cell cycle inhibitor p21 and p53s15 on tumors sampled one day after last injection. Individual bands (S1, S2, S3) correspond to 3 independent tumor samples per group of treatment.

This antitumor efficacy was characterized on tumors sampled one day after the last injection (D41). Histological studies (Figure 6A) showed a significant decrease in mitotic index in tumor tissues from **8-QA**-treated mice (9.7 ± 9.0 mitosis per 10 fields) compared to controls (40.8 ± 18.2 mitosis per 10 fields) (p = 0.033). Next, western blot evidenced a significant increased of phosphorylated-p53 (S15), leading to an induction of p21 observed only in **8-QA** treated tumors (Figure 6B).

### *In vivo*, 8-QA induced DNA damages in hypoxic areas

**Figure 7:**
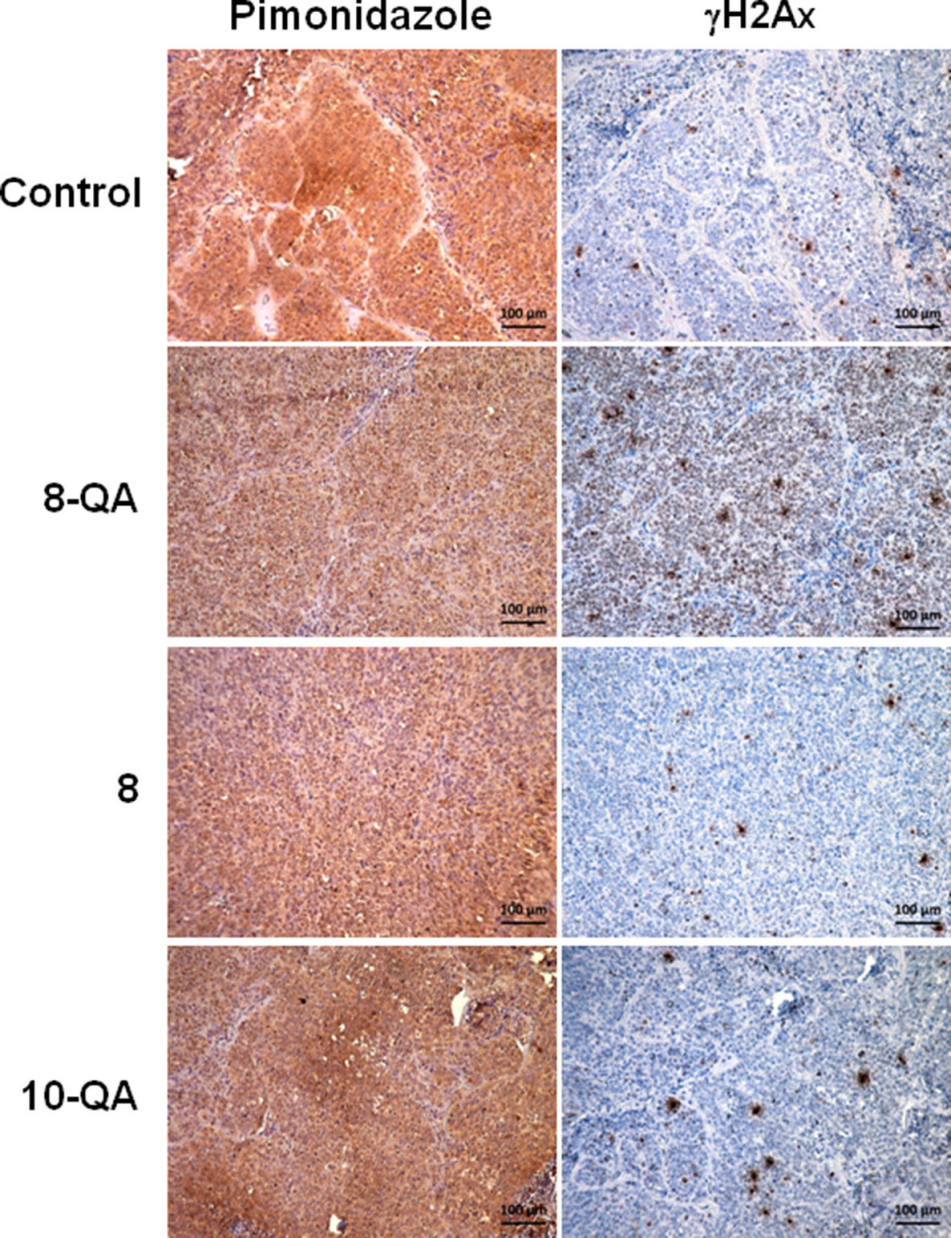
Distribution of DNA damages in treated tumor tissue Representative images of immunostaining of hypoxia marker pimonidazole (brown staining) and DNA damage (γH2AX) marker on two consecutive tumor sections. Pimonidazole was i.p.-injected at 60 mg/kg 1 hour before mice were euthanized.

To provide further evidence that the antitumor activity of **8-QA** was mainly localized to the hypoxic environment, we performed immunostaining investigations on two successive slices using DNA damage marker γH2Ax and hypoxia marker pimonidazole. In the **8-QA** treated group, a strong increase of γH2AX-positive cells was found in the pimonidazole-positive hypoxic areas (Figure 7). Interestingly, lower γH2Ax staining was observed in pimonidazole-negative normoxic areas (Supplementary Figure 1). As expected, derivatives **8** and **10-QA**, that have no antitumor efficacy, did not cause any γH2AX-positive cells in tumor hypoxic areas (Figure 7).

To confirm that the increased of γH2Ax staining, observed in **8-QA** treated tumors, was not due to the blocking of cells in S phase, we analyzed on treated tumor samples, phosphorylated CHK-1, which is known to be ATR dependent. As expected, no increase of p-CHK1 was observed in **8-QA** treated samples, suggesting that γH2Ax increase observed in immunostaining could not be attributed to an ATR-dependent replicative stress (Supplementary Figure 2).

### 8-QA reduces hematological side effects and does not alter healthy articular cartilage

**Figure 8:**
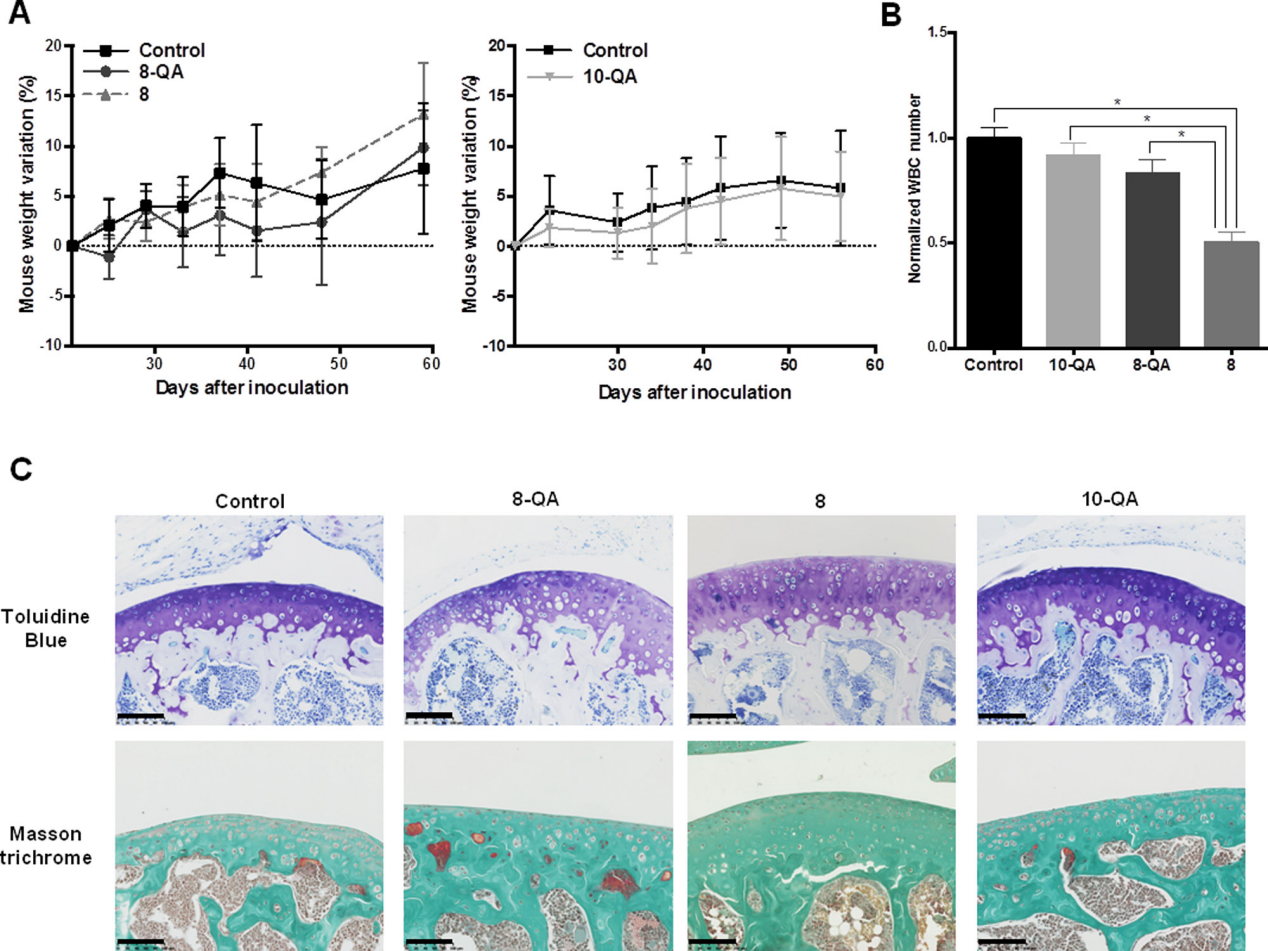
Side-effect evaluations **(A)** Weight variation from Day 20 post-inoculation for **8** and **8-QA**-treated mice (on the left) and for **10-QA** treated mice (on the right). Data are represented as mean ± SD. **(B)** White Blood Cell (WBC) count (n≥4 per group of treatment) normalized to the control groups the day after last dose. Blood was sampled by retro-orbital puncture. Data are represented as mean ± SD. **(C)** Anatomopathological analysis of healthy cartilage from mouse knee joints sampled 24 hours after the last dose (n = 5 per group of treatment). Articular cartilages were stained by Toluidine blue and Masson trichrome to evidence proteoglycans and collagen respectively. Scale bar = 100 μm.

Side effects associated with HAP treatments were assessed by monitoring mouse weight and by hematological analyses performed 24 hours after the last dose. Derivatives **8-QA**, **8** and **10-QA** did not cause any weight loss compared to controls during the treatment (Figure 8A). Hematological analyses found that non-QA conjugated derivative **8** caused a significant decrease in white blood cell count compared to controls (p < 0.001), QA-conjugated derivatives **8-QA** (p = 0.004) and **10-QA** (p = 0.003) (Figure 8B). These results also confirm the potential of a QA vectorization strategy to avoid the side effects commonly associated with chemotherapy.

Given that articular cartilage is also an ECM-rich tissue, we documented potential unexpected side effects on the macromolecules responsible for joint functionality, i.e. collagens and proteoglycans. As illustrated in Figure 8C, we found no evidence of fibrillation and no alteration of cartilage integrity with QA-derivative treatments, as confirmed by homogeneous Masson trichrome staining of collagen and toluidine blue staining of proteoglycans.

## DISCUSSION

The therapeutic management of chondrosarcoma is highly challenging, with *en bloc* resection with wide surgical margins being the only effective treatment [2, 3]. Complement adjuvant therapies are needed when wide resection is not possible, particularly in critical anatomic locations such as the pelvis, the axial skeleton, and also in many cases of local recurrence [27].

Nevertheless, the large amount of ECM combined with the poor vascularity of chondrosarcoma tumors means that the anticancer agents have to diffuse over a relatively long distance in order to reach tumor cells and be effective.

Our hypothesis is that the best approach for a personalized therapy for chondrosarcoma could be exploit its phenotypic microenvironment features, mainly chondrogenic ECM and hypoxia.

As with all molecular targeted therapies, the therapeutics vectorized to hypoxic and acidic microenvironments can only be effective if they physically reach their targets. As delivery and retention of drugs in the tumor is influenced by the complex microenvironment, there is an ongoing challenge to improve the overall delivery of therapeutics to the general tumor areas in order to improve targeting selectivity. The physical and chemical hallmarks of the tumor microenvironment can therefore open new ways to design more rational and effective therapeutic regimens, and potentially with less side effects compared to traditional therapies [28].

Our team working on ECM-targeting approaches has patented the use of a proteoglycan-targeting strategy that uses a quaternary ammonium (QA) function as ligand [12]. Proof-of-concept for therapeutic application was demonstrated for the QA-conjugated melphalan that increased therapeutic index compared to non-QA-conjugated melphalan [29]. Building on this result, we developed a QA conjugate of a phosphoramidate mustard bearing a nitroimidazole as the hypoxia sensitive function, named **8-QA**, which releases the DNA crosslinking mustard agent under hypoxic conditions. The efficacy of this compound was compared *in vitro* and *in vivo* against its non-QA equivalent **8** and the non-cleavable compound **10-QA**.

First, we validated the affinity of the QA-derivatives only, i.e. **8-QA** and **10-QA** for aggrecan by SPR. As non-QA derivative **8** showed no affinity, these results validate the QA entity for aggrecan binding.

We then tested the selective cytotoxicity profile of **8-QA** on HEMC-SS cells *in vitro*. As expected, **8-QA** enhanced cytotoxicity under hypoxic conditions and induced a high accumulation of HEMC-SS cells in S-phase and in apoptosis. However, the dose response effect of **8-QA** observed in normoxic condition for cell cycle analyses, was lost in hypoxia. The lack of this dose response in hypoxia can be explain by the fact that the cytostatic activity of **8-QA** was maximal from 25 μM. Indeed, the IC_50_ value of **8-QA** on HEMC-SS cells after 24 hours of treatment was 29.3 ± 8.0 μM in normoxic conditions while it was only 4.3 ± 1.2 μM in hypoxia. Consequently, we hypothesized that from 25 μM, a sufficient quantity of **8-QA** was cleaved by the hypoxic conditions to have a maximal cytostatic activity.

As expected, non-cleavable compound **10-QA** had no effect up to 200 μM.

We had previously investigated the cytotoxic activity of **8-QA** on human osteosarcoma Saos-2 cells (unpublished data). Interestingly, **8-QA** showed only weak cytotoxic activity on Saos-2 cells in hypoxic conditions (IC_50_ of 131.5 ± 29.0 μM), thus confirming the cytotoxic specificity of QA-alkylating agent conjugates for chondrosarcoma cells [14].

Realizing the limitations of monolayer culture, and inspired by the complexity of the native tumor microenvironment, researchers have developed various 3D models that recapitulate some of the features of solid tumor tissues, such as gradient distribution of chemical and biological factors, expression of pro-angiogenic and MDR proteins, or dynamic and reciprocal interactions between the tumor and its stroma. The HEMC-SS spheroid culture model developed here reproduces the proteoglycan-rich ECM of chondrosarcoma and the hypoxic environment found in tumor tissue *in vivo*. This model increased HAP treatment resistance, as evidenced by higher IC_50_ value for compounds 8-QA and 8 than those obtained on monolayer cultures, as commonly observed in the literature [30]. Previous studies have also demonstrated that the penetration of drug (i.e Doxorubicin) was restricted to the outer few cell layers of larger breast spheroids and effectiveness was inversely proportional to distance from the periphery [31]. Note that non-QA derivative **8** has a far higher cytotoxic activity *in vitro* in hypoxic HEMC-SS spheroids than **8-QA**. This raises the question of the role of QA function in prodrug uptake and penetration into the deeper layers of spheroids. However, the role of QA for tumor targeting was clearly demonstrated *in vivo*, since non-QA derivative **8** did not show antitumor efficacy.

Analysis of the antitumor effect of the derivatives on hypoxic and chondrogenic HEMC-SS xenografts showed that **8-QA** caused significant inhibition of tumor growth with a TGI value of 62.1% the day after the last injection (D41). Antiproliferative activity was confirmed by a significant increase of cell cycle inhibitor p21 and apoptotic marker p53S15 for **8-QA**-treated tumors. The co-localization of DNA damage marker γH2AX-positive immunostains in the pimonidazole-positive hypoxic regions of xenograft tumors is consistent with the selective targeting of hypoxic compartments of tumors by **8-QA**.

Interestingly, **8-QA** did not induce any side effects whereas non-QA derivative **8** was associated with hematological side effects. These results confirmed the high potential of proteoglycan-targeting by QA as a strategy to improve therapeutic index.

Selective targeting of hypoxic tumor regions with HAP has been demonstrated as a promising therapeutic strategy for the treatment of cancers. Indeed, TH-302, a 2-nitroimidazole-conjugated prodrug of bromo-isophosphoramide mustard (Br-IPM) demonstrated a broad anti-tumor activity across a panel of human tumor xenografts, including osteosarcoma, with clear evidence of selective eradication of hypoxic cells and neighboring cells via the bystander effect [32, 33]. In combination regimens with cytotoxic drugs, targeted therapeutics or radiotherapy, TH-302 has been shown to enhance antitumor activity and reduce the ability of hypoxic cells to repopulate tumors following re-oxygenation [32–39]. To our knowledge, no HAP strategy has yet been described for chondrosarcoma which is characterized by overexpression of hypoxia marker HIF-1α that may play an important role in the prognosis of this tumor [40].

To our knowledge, by conjugating QA to 2-nitroimidazole phosphoramidate mustard, we are the first to propose a HAP strategy for chondrosarcoma. Indeed, compound **8-QA** improved the selectivity of HAP in terms of antitumor efficacy and adverse side effects.

Given that proteoglycans play an important role in cell–cell and cell–ECM interactions and signaling in a variety of cellular functions (motility, adhesion, growth) [41, 42], targeting negatively-charged GAG by a positively-charged QA entity could alter the electrical and physical properties of ECM-like osmotic pressure, resulting in a process that remodels the tumor microenvironment involved in tumor regression [28, 43].

Furthermore, although the QA entity is expected to selectively address to the proteoglycan-rich ECM of chondrosarcoma, the *in vivo* kinetics of phosphoramidate mustard release and DNA alkylation need to be determined to elucidate the precise molecular mechanism involved.

In conclusion, our results in HEMC-SS models can be considered as a first strong proof of concept for proteoglycan-targeting by HAP as a new therapeutic strategy for chondrosarcoma. Further investigations on additional chondrosarcoma cell lines will be conducted to assess the potentiality of this novel derivative **8-QA** (that will be named ICF05016), including in combination regimens with conventional chemotherapeutics or radiotherapy.

## MATERIALS AND METHODS

### Chemical synthesis

Target compound **8-QA** was synthesized as depicted in Figure 1A *via* phosphorylation of the key intermediate 2-nitroimidazole **6**. In order to determine the contribution of the ligand moiety to *in vitro* and *in vivo* activities, the derivative **8** was designed and prepared for comparison with **8-QA**. In addition, to assess the importance of the release of the phosphoramidate mustard after hypoxia-cleavage of the prodrug, the non-cleavable compound **10-QA** was also synthesized with the mustard tethered to a benzyl group.

Briefly, synthesis of 5-hydroxymethyl-1-*N*-methyl-2-nitro-1*H*-imidazole **6** was carried out as depicted in Figure 1A, adapted from a process patented by Matteucci *et al* [21], starting from sarcosine ethyl ester **1**. The synthesis of 1-methyl-2-nitroimidazole derivatives has proven challenging, as reported recently by O’Connor *et al* [22, 23]. First, sarcosine ethyl ester **1** was *N*-formylated by treatment with ethylformate in presence of potassium carbonate. A one-pot 3-step procedure was then used to yield 2-aminoimidazole **3**: (i) *C-*formylation of the intermediate **2** at the α-carbon was achieved by treatment with sodium hydride and ethylformate; (ii) removal of the unwanted formyl group on the secondary amine was performed under acidic conditions at reflux; (iii) subsequent Marckwald cyclization with cyanamide under reflux in aqueous acetate-buffered conditions afforded the desired 2-aminoimidazole **3** in 50–60% yields. Then, diazotization of the amino group with an aqueous solution of sodium nitrite using the procedure reported by Matteucci *et al.* afforded compound **4** in 75% yield. The ester moiety of this intermediate was then successfully reduced using two different protocols. The first protocol involved a two-step pathway *via* the corresponding carboxylic acid **5** which was obtained in 77% yield by hydrolysis of ester **4** under mild basic conditions and then activated by isobutylchloroformate before reduction using sodium borohydride to afford alcohol **6** in 75% yield. The second strategy consisted in directly reducing ester **4** to alcohol **6**, in 70% yield, using *in situ* formation of lithium borohydride. This second option is more interesting in terms of yield and time.

Phosphorylation of the key 2-nitroimidazole intermediate **6** to give compounds **7** and **8** was then carried out using a modified version of Hernick *et al*'s procedure [24]. Briefly, the lithium alkoxide of **6** was formed using lithium bis(trimethylsilyl) amide and reacted with bis(2-chloroethyl)phosphoramidic dichloride [25]. This step was followed up by phosphorus NMR. The corresponding propylamine derivative was then added in the reaction mixture to afford **7** or **8** in 48% and 65% yields, respectively. Quaternization of the tertiary amine of **7** was achieved using methyl iodide, leading to quaternary ammonium derivative **8-QA** in quantitative yield. A similar procedure was used to obtain compounds **9** and **10-QA**, except for the base used to generate the alkoxide. In this case, lithium bis(trimethylsilyl)amide was replaced by sodium hydride to lead to sodium benzylate, as depicted previously in the literature [26], which was directly engaged in the phosphorylation reaction with bis(2-chloroethyl)phosphoramidic dichloride.

### Surface plasmon resonance (SPR) binding assays

SPR assay was carried out on a Biacore T200 instrument (GE Healthcare) with a CM4 sensor chip (GE Healthcare). To immobilize aggrecan from bovine articular cartilage (Sigma-Aldrich), the sensor chip was first activated using a 1:1 mixture of 0.2 M 1-ethyl-3-(3-dimethylaminopropyl)carbodiimide hydrochloride and 0.5 M *N*-hydroxysuccinimide (amine coupling kit, GE Healthcare) at a flow rate of 5 μL/min for 10 minutes. Aggrecan was then coated on the sensor chip at 400 μg/mL in running buffer HBS-P^+^ 1X (0.01 M HEPES, 0.15 M NaCl, 0.05% v/v surfactant p20, pH 7.4) with 6 mM hexadecyltrimethylammoniumbromide (CTAB) at 5 μL/min to a level of ~500 response units. Unoccupied binding sites were blocked using 1 M ethanolamine (pH 8.5) (Amine coupling kit, GE Healthcare) at 5 μL/min during 10 minutes. The studied molecules (analytes) were diluted in running buffer HBS-P^+^ 1X before application to the sensor chip at 30 μL/min. Regeneration of the sensor chips was achieved using 2 M NaCl solution during 150 seconds. Dissociation constants (K_D_) were determined using Biacore T200 Evaluation software with a “steady-state affinity analysis” after injection of five different concentrations of analytes.

### Monolayer and spheroid cultures of the human HEMC-SS chondrosarcoma cell line

Human HEMC-SS chondrosarcoma cell line was obtained from the European Collection of Authenticated Cell Cultures and cultured in DMEM/F12 medium (Life Technologies) supplemented with 10% fetal calf serum (Dutscher) and 4 μg/mL gentamicin.

To generate HEMC-SS spheroids, cells were harvested from culture flasks by trypsinization and seeded in 96-well non-adherent plates (Nunc) with 100 μL of 0.5% methylcellulose (Bio-Techne) diluted in the corresponding medium at a density of 1000 cells *per* well. Cultures were maintained in a 5% CO_2_ humidified environment at 37°C.

Spheroid cell numbers were determined at different times (Day 1 to Day 10) after cell dissociation by incubation with Trypsin-EDTA (Life Technologies) for 5 minutes at 37°C.

### Scanning electron microscopy (SEM) analysis of spheroid structure

HEMC-SS spheroids (Day 10) were fixed with 1.6% glutaraldehyde in 0.2 M sodium cacodylate buffer (pH 7.4) overnight at 4°C, then washed three times with 0.2 M sodium cacodylate buffer (pH 7.4). For secondary fixation, spheroids were immersed in 1% osmium tetroxide in 0.2 M sodium cacodylate buffer (pH 7.4). Fixed spheroids were subsequently dehydrated with a graded ethanol series (25, 50, 75, 95, and 100%), immersed in hexamethyldisilazane (HMDS) for 10 minutes (two times) at RT. The spheroids were freeze-dried until the HMDS evaporated, then mounted, coated with palladium gold, and observed under a scanning electron microscope (JEOL) at an acceleration voltage of 5 kV.

### SOX-9 real-time quantitative RT-PCR

Total RNA was extracted from 5 × 10^6^ HEMC-SS cells or approximately 180 spheroids at Day 7, Day 10 or Day 14 post-seeding, using the Nucleospin® RNA isolation kit (Macherey-Nagel). After quantification with Nanodrop (Multiskan GO, Thermo Scientific), 100 ng of total RNA were reverse-transcribed in cDNA using the Thermoscript RT-PCR kit (Thermo Scientific). Real-time qPCR was carried out on cDNA diluted at 1/100 using Kapa SYBR® Fast qPCR master mix (Kapa Biosystems). The following sense and anti-sense human primers were used: SOX-9 5’-TCGCTCTCGTTCAGAAGTCTC-3’ and 5’-GTACCCGCACTTGCACAAC-3’; GAPDH 5’-CCTCCAAGGAGTAAGACCCC-3’ and 5’-TGTG AGGAGGGGAGATTCAG-3’. Relative quantification of SOX-9 was performed using the standard curve method by normalizing to housekeeping gene GAPDH.

### Spheroid hypoxia analysis using a LOX-1 hypoxia probe

The LOX-1 hypoxia probe (SCIVAX) is a phosphorescent light-emitting iridium complex that is oxygen-quenched: its phosphorescence decreases in response to increasing oxygen concentration. LOX-1 was added to the spheroid culture medium at a final concentration of 200 μM 24 hours before fluorescent detection using Axioplan microscopy (Zeiss).

### *In vitro* proliferation assay

After trypsinization, HEMC-SS cells were seeded in 96-well plates at a density of 20.10^3^ cells in 150 μL of the corresponding culture medium and allowed to adhere overnight. For 3D cell culture, Day 10 spheroids were used. Increasing drug concentrations diluted in DMSO (maintaining final DMSO concentration at 0.5% (v/v)) were added. After plate incubations for 24 hours in normoxic (21% O_2_, 5% CO_2_, 37 °C) or hypoxic (N_2_, O_2_ < 0.3%, 37 °C) conditions, the culture media were removed and 2D and 3D cells were washed with PBS and left to grow for 48 hours in normoxic conditions. Viability of cells cultured in monolayer or spheroids was quantified by AlamarBlue assay. Cytotoxic activity was expressed as the drug concentration that inhibited cell growth by 50% (IC_50_). Experiments were performed in triplicates. Data are represented as means ± SD. Statistical significance was determined using one-way ANOVA followed by Tukey's post-hoc multiple comparisons test. Results were considered significant at *p* < 0.05.

### Cell cycle determination and apoptosis evaluation

At 24 hours before treatment, 1.10^6^ HEMC-SS cells were seeded in 6-well plates (Nunc) at 3 mL/well of corresponding culture media. Cells were treated with **8-QA** compound at 25 μM, 50 μM, or 100 μM final concentrations for 24 hours, under either normoxic (21% O_2_, 5% CO_2_, 37°C) or hypoxic (N_2_, O_2_ < 0.3%, N_2_, 37°C) conditions. After incubation, the cells were harvested by trypsinization. The cell suspension containing both floating and adherent cells was centrifuged (400 *g*, 8 min at +4°C) in PBS. Cell cycle analyses were carried out by resuspending cells in 500 μL of ribonuclease 1 (1 mg/mL) added with 500 μL of propidium iodide (1 mg/mL) (Sigma Aldrich), and apoptotic cells were detected using the Annexin V-FITC kit (Miltenyi Biotec). The cell cycle distribution and proportion of apoptotic cells were analyzed by flow cytometrey (LSR II flow cytometer, BD Biosciences). Experiments were performed in triplicates. Data are represented as means ± SD. For cell cycle analyses, statistical significance was determined using two-way ANOVA followed by Tukey's post-hoc multiple comparison test. For Annexin V test, significance was determined using two-way ANOVA followed by Bonferroni's post-hoc multiple comparison test.

Results were considered significant at *p* < 0.05.

### Xenograft model

*In vivo* studies were carried out on 50 female Severe Combined ImmunoDeficiency (SCID) mice aged 4–5 weeks old (Janvier Labs). All animal studies were carried out in accordance with the directive 2010/63/EU after approval by the institutional review board (C2E2A, authorization #CE85-12).

To obtain *in vivo* tumors, 10^7^ HEMC-SS cells in 100 μL of PBS were injected with 100 μL of Matrigel® (BD Biosiences) subcutaneously into the right flank of the mice.

### *In vivo* imaging of tumor hypoxia and vascularization by photoacoustic and Doppler imaging

*In vivo* imaging was performed on 4 representative mice. Anesthetized mice (1.5% isoflurane) were placed supine on a thermostatically-controlled heating pad with the paws tied to the table through ECG electrodes. A colorless aqueous warmed ultrasonic gel (Supragel®) stripped of any air bubbles was applied between the skin and the transducer. Ultrasound and photoacoustic imaging were performed using a transducer with a 21 MHz central frequency and the VEVO LAZR system (FUJIFILM Visualsonics Inc), with 3D scans recorded digitally. To determine tumor volumes, tumor areas in coronal planes were measured by manually delineating margins using Vevo®LAB 1.7.2 software that calculated the corresponding volume on each coronal slice. Global tumor hypoxia was investigated by photoacoustic imaging in OxyHemo-Mode to determine the average values of oxygen perfusion (SO_2_). For vascularization assessment, Doppler imaging was used to monitor the tumors. Tumor perfusion status was assessed by contrast-enhanced ultrasound (CEUS) imaging after an i.v. injection of 50 μL of Vevo MicroMarker™. Data were processed using the VevoCQ™ software.

### *In vivo* antitumor efficacy

The antitumor efficiency of **8-QA** was first studied respectively to its non-QA equivalent (**8**) used as negative control for proteoglycan targeting. In a second independent study, we evaluated the antitumor activity of non-hypoxia activated derivative (**10-QA**) used as negative control of hypoxia activation, respectively to another control group. For both studies, at 21 days post-cell inoculation mice were randomized into three or two groups (10–12 animals per group). Treatment protocol consisted in 6 doses at 4-day intervals injected by i.v route: **8-QA** (47.0 μmol/kg) **and 10-QA** (46.7 μmol/kg)were injected at 50% of Maximum Tolerated Dose (MTD) while non-targeted derivative **8** (47.0 μmol/kg) was injected at equimolar dose of **8-QA**. All molecules were diluted in saline with 2% ethanol. Control animals received vehicle according to the same schedule.

Tumor volume (TV) was calculated using the following formula:

TV (mm^3^) = [length x (width)^2^]/2.

Tumor Growth Inhibition (TGI) was calculated as follows: TGI (%) = [1-(Tn/Cn)] × 100, where Tn is the tumor volume of the treated group at day n and Cn the tumor volume of the control group at day n.

The day after the last dose, 50% of animals of each group were euthanized by CO_2_ inhalation, and tumors were sampled for histology and immunohistochemistry analyses (n≥3 tumors samples per group of treatment).

To assess side effects, mouse weight was monitored and pathology analyses on healthy articular cartilage and hematological analyses were carried out at 24 hours after the last injection. Approximately 50 μL of blood, obtained by retro-orbital sampling, were analyzed on a Melet Schloesing MS9-5 Hematology Analyzer (n = 5 blood samples per group of treatment) (Diamond diagnostics).

Data are represented as means ± SD. For antitumor efficiency and mouse weight monitoring, statistical significance was determined using two-way ANOVA. For tumor weight, mitosis number and hematological analyses, statistical significance was determined using one-way ANOVA. Both tests were followed by Tukey's post-hoc multiple comparisons test. Results were considered significant at *p* < 0.05.

### Western Blot analysis

Approximately 30 μg of proteins from tumors (n = 3 independent tumor samples per group of treatment) recovered one day after last dose were loaded onto 10% acrylamide gels and separated by SDS-PAGE. Proteins were then transferred to nitrocellulose membranes (Millipore) and incubated with specific primary antibodies p53S15 (Cell Signaling), p21 (Santa Cruz), p-Chk1 (S345) (Cell Signaling) or GAPDH (Santa Cruz) used as housekeeping protein. Antibody-antigen interaction was evidenced by chemiluminescence (Amersham) using HRP-conjugated secondary antibodies (Southern Biotech). Signal was detected and quantified using Image Lab software (BioRad). Data are represented as means ± SD. Statistical significance was determined using one-way ANOVA followed by Tukey's post-hoc multiple comparisons test. Results were considered significant at *p* < 0.05.

### Histology and immunohistochemistry on spheroids and tumors

For tumors, the exogenous hypoxia marker pimonidazole hydrochloride (Hypoxyprobe™) was i.p.-injected at 60 mg/kg one hour before mice were euthanized. Tumors were removed, fixed in 10% neutral buffered formalin, and embedded in paraffin. For spheroid cultures, pimonidazole was added at a final concentration of 200 μM two hours before fixation and paraffin-embedding.

Five μm-thick sections were cut and adhered to poly-L-Lysine-coated glass microscope slides. After deparaffinization and rehydration, slides were stained by haematoxylin–eosin (H&E) or Alcian blue (pH 1) to visualize aggrecan or used for immunohistochemistry. After antigen retrieval, endogenous peroxidase quench and saturation, slides were incubated with the primary antibody: anti-Ki 67 (Abcam) rabbit polyclonal antibody (1/2000, 11 hours, RT), anti-pimonidazole (Hypoxyprobe™) mouse monoclonal antibody (1/100, 30 minutes, RT) or anti-phospho-histone-H2AX (Merck Millipore) mouse monoclonal antibody (1/2000, 11 hours, RT). HRP-conjugated rabbit anti-mouse IgG1 secondary antibody (Hypoxyprobe, 1/100, 30 minutes, RT) was used to detect pimonidazole adduct and a biotinylated goat anti-mouse IgG1 secondary antibody (Vector Laboratories, 1/500, 1 hour) was used to detect anti-Ki-67 and anti-phospho-histone-H2AX. Biotin was then complexed with streptavidin-coupled HRP (Vector Laboratories, 1/500, 30 minutes). HRP was detected using a DAB ultravision detection system (Thermo Scientific).

### Articular cartilage staining

Mouse paws (n = 5 per group of treatment) were fixed in 10% neutral buffer formalin, decalcified with 4.13% EDTA and 0.2% paraformaldehyde in PBS for 96 hours using KOS microwave tissue processor (Milestone), and embedded in paraffin. Then, 4–5-μm thick sections were stained by Toluidine Blue (pH 2.5) to visualize aggrecan or by Masson trichrome to visualize collagen. All images were captured with NanoZoomer (Hamamatsu).

PRT-k, Translational Cancer Research: INCa-DGOS, D-TECT project reference R15066CC / RPT15003CCA

## SUPPLEMENTARY MATERIALS FIGURES


