# Supplementary material for: Proteoglycan-targeting applied to hypoxia-activated prodrug therapy in chondrosarcoma: first proof-of-concept
Source: Oncotarget. 2017 Sep 27;8(56):95824–40. doi: 10.18632/oncotarget.21337 (PMC5707064; doi:10.18632/oncotarget.21337)
Supplement: Supplementary file 1 [file oncotarget-08-95824-s001.pdf]

Proteoglycan-targeting applied to Hypoxia-Activated Prodrug therapy in chondrosarcoma: First proof-of-concept

SUPPLEMENTARY MATERIALS

MATERIALS AND METHODS, CHEMISTRY

All commercially available reagents and solvents were purchased at the following commercial suppliers: Sigma Aldrich (Saint-Quentin Fallavier, France), Acros Organics (Geel, Belgium), Fisher Scientific (Illkirch, France), Carlo Erba Reagents (Val de Reuil, France), VWR (Fontenay-sous-Bois, France) and Alfa Aesar (Karlsruhe, Germany) and were used without further purification. All solvents were dried using common techniques. Air and moisture sensitive reactions were carried out under dry argon atmosphere. Analytical thin-layer chromatography (TLC) was performed on precoated silica gel aluminium plates (60 F_{254}, 0.2 mm thick) (Merck or SDS) using the indicated solvent mixture expressed as volume/volume ratios. The plates were visualized with ultraviolet light (254 nm) and (or) by development with ninhydrine or a NBP reagent (Determination of alkylating agents, NBP 2.5% in acetone then KOH 10% in MeOH). Column chromatography was performed on silica gel 60A normal phase, 35–70 µm (Merck or SDS). Uncorrected melting points (mp) were measured on an electrothermal capillary Digital Melting Point Apparatus (IA9100, Bibby Scientific, Roissy, France). Infrared spectra (IR) were recorded in the range 4000–440 cm^{-1} on a IS10 with attenuated total reflectance (ATR) accessory Nicolet (Fisher Scientific). ^1H NMR and ^{13}C NMR spectra (500 or 200 MHz for ^1H and 50 MHz for ^{13}C) were recorded on a Bruker Avance 500 or a Bruker Avance 200 instrument; ^{31}P NMR spectra (202 MHz) were recorded on a Bruker Avance 500 apparatus using H_3PO_4 (1%) in D_2O as internal reference (0 ppm). Chemical shifts are reported in parts per million (ppm) and coupling constants (*J*) are given in Hz. To describe spin multiplicity, standard abbreviations such as s, d, dd, t, q, qt, st, td, m, br.s, br.t. referring to singlet, doublet, doublet of doublet, triplet, quartet, quintet, sextet, doublet of triplet, multiplet, broad singlet, broad triplet respectively, are used. Compounds were analyzed by High-Resolution Mass Spectrometry in positive mode (HRMS, Waters® Micromass® Q-Tof micro™ Mass Spectrometer, UBP-START, Blaise Pascal University, Clermont-Ferrand, France).

Sarcosine ethyl ester hydrochloride salt (1)

To an ice-cold solution of sarcosine (20 g, 224 mmol) in ethanol (100 ml) was added dropwise thionyl chloride (120 ml), while maintaining temperature around -10 °C. Then, the solution was heated to 55 °C for 2 h. Solvent and traces of thionyl chloride were removed by evaporation under reduced pressure. The residue was dried in a desiccator overnight to afford compound **1** (32.4 g, 212 mmol) as a white powder, which was used in the next step without further purification. Yield 85%; mp 112 °C (Lit. mp 115 °C) [1]; IR (ATR) ν 2970-2440, 1742, 1229 cm^{-1}; ^1H NMR (200 MHz, $CDCl_3$) δ 9.66 (br.s, 2H, $N\underline{H}_2^+$), 4.24 (q, 2H, ^3J = 7.1 Hz, $C\underline{H}_2CH_3$), 3.84 (br.t, 2H, ^3J = 5.5 Hz, $NH_2^+C\underline{H}_2$), 2.80 (br.t, 3H, ^3J = 5.5 Hz, $C\underline{H}_3NH_2^+$), 1.27 (t, 1H, ^3J = 7.1 Hz, $CH_2C\underline{H}_3$); ^{13}C NMR (50 MHz, $CDCl_3$) δ 166.1 ($\underline{C}O_2$), 62.65 ($\underline{C}H_2CH_3$), 48.9 ($NH_2^+\underline{C}H_2$), 33.3 ($NH_2^+\underline{C}H_3$), 14.0 ($CH_2\underline{C}H_3$).

Ethyl *N*-formylsarcosinate (2)

Sarcosine ethyl ester hydrochloride salt **1** (31 g, 203 mmol) was dissolved in ethanol (130 ml) and potassium carbonate (28 g, 202 mmol) and ethyl formate (130 ml) were added. The solution was stirred at room temperature overnight. After filtration and washing with ethanol (40 ml), the filtrate was concentrated and diluted with water (300 ml). The residue was extracted with ethyl acetate (5 × 200 ml). The organic extracts were combined, dried over magnesium sulphate, filtered and concentrated under reduced pressure to give ethyl *N*-formylsarcosinate **2** (27 g, 186 mmol) as a yellow liquid. Yield 92%; ^1H NMR (200 MHz, $CDCl_3$) δ (two rotamers) 8.06, 7.99 (2s, 1H, CHO), 4.16, 4.18 (2q, ^3J = 7.1 Hz, 2H, OCH_2), 4.04, 3.94 (2s, 2H, NCH_2), 2.99, 2.88 (2s, 3H, NCH_3), 1.27, 1.23 (2t, ^3J = 7.1 Hz, 3H, $OCH_2C\underline{H}_3$). These data are in good agreement with the literature values [2].

Ethyl 2-amino-1-*N*-methyl-1*H*-imidazole-5-carboxylate (3)

To a solution of ethyl *N*-formylsarcosinate **2** (24 g, 165 mmol) in ethyl formate (85 ml) cooled in an ice-water bath, was carefully added in several portions sodium hydride (60 wt% in mineral oil, 7.6 g, 190 mmol). After addition was completed and hydrogen release stopped, the temperature was allowed to rise gradually to room temperature and stirring continued overnight. After concentration, the residue was triturated with hexane (4 × 70 ml) and dissolved in a mixture of ethanol and concentrated hydrochloric acid (50/30 ml) before heating to 110 °C for 1 h. The reaction mixture was cooled down, filtered, the residue washed with ethanol (50 ml) and the

filtrate concentrated under reduced pressure to yield a thick brown oil. The residue was suspended in an aqueous solution of 10% acetic acid (90 ml), before addition of sodium acetate (38 g, 461 mmol) and cyanamide (15 g, 351 mmol). The solution was stirred at 95 °C for 1 h and concentrated to approximately 1/3 of its original volume. After cooling in an ice-water bath, the pH was adjusted to about 9 by addition of an aqueous saturated sodium carbonate solution. The yellow precipitate formed was filtrated and washed with ethyl ether to afford compound **3** (13.7 mg, 81.1 mmol). The filtrate was then extracted with ethyl acetate (3 × 200 ml), the combined organic layers dried over magnesium sulphate, filtered, and concentrated under reduced pressure. The resulting residue was purified by column chromatography on silica gel (ethyl acetate/ethanol, 95/5, v/v) to yield an additional batch of compound **3** (1.42 g, 8.39 mmol) as a yellow solid. Yield 55%; mp 163 °C (Lit. mp 130-133 °C) [3]; Rf 0.33 (SiO_2, ethyl acetate/ethanol, 9/1, v/v); ^{1}H NMR (200 MHz, $CDCl_3$) δ 7.43 (s, 1H, C<u>H</u>), 4.59 (br.s, 2H, N$\underline{H}_2$), 4.25 (q, 2H, ^3J = 7.1 Hz, C$\underline{H}_2$$CH_3$), 3.66 (s, 3H, NC$\underline{H}_3$), 1.32 (t, 3H, ^3J = 7.1 Hz, CH_2C$\underline{H}_3$). These data are in good agreement with the literature values [3, 4].

Ethyl 1-*N*-methyl-2-nitro-1*H*-imidazole-5-carboxylate (4)

To a solution of sodium nitrite (18.3 g, 266 mmol) in water (55 ml) cooled around -5 °C in an ice-salt bath, was added dropwise a solution of the amino ester **3** (6.42 g, 38.0 mmol) in acetic acid (42 ml). The temperature was allowed to rise gradually to room temperature and the reaction mixture was stirred overnight. The reaction mixture was extracted with dichloromethane (3 × 50 ml). The combined organic layers were dried over magnesium sulphate, filtered and evaporated under reduced pressure. The residue was purified by column chromatography on silica gel using cyclohexane/ethyl acetate (7/3, v/v) as eluent to yield nitro ester **4** as yellow crystals (5.67 g, 28.5 mmol). Yield 75%; mp 60-62 °C (Lit. mp 56-58 °C [3] and 65-66 °C [5]); Rf 0.40 (SiO_2, cyclohexane/ethyl acetate, 7/3, v/v);^{1}H NMR (500 MHz, $CDCl_3$) δ 7.73 (s, 1H, C<u>H</u>), 4.39 (q, 3H, ^3J = 7.1 Hz, C$\underline{H}_2$$CH_3$), 4.34 (s, 3H, NC$\underline{H}_3$), 1.40 (t, 3H, ^3J = 7.1 Hz, CH_2C$\underline{H}_3$). These data are in good agreement with the literature values [3, 4].

1-*N*-Methyl-2-nitro-1*H*-imidazole-5-carboxylic acid (5)

A suspension of ethyl 1-methyl-2-nitro-1H-imidazole-5-carboxylate **4** (11.7 g, 58 mmol) in a sodium hydroxide solution (1 M, 200 ml, 200 mmol) was stirred at 20 °C overnight. The solution was cooled and pH of the solution was adjusted to 1 by dropwise addition of an aqueous concentrated hydrochloric acid solution. The mixture was extracted with ethyl acetate (7 × 90 ml). The combined organic fractions were dried over magnesium sulphate, filtered and the solvent evaporated under reduced pressure to give carboxylic acid **5** (7.65 g, 44.7 mmol) as a white powder, which was used in the next step without further purification. Yield 77%; mp 159-160 °C (lit. mp 160–161 °C) [2]; Rf 0.36 (SiO_2, cyclohexane/ethyl acetate, 7/3, v/v); ^{1}H NMR (200 MHz, acetone-d_6) δ 7.73 (s, 1H, C<u>H</u>), 4.32 (s, 3H, NC$\underline{H}_3$). These data are in good agreement with the literature values [2].

5-Hydroxymethyl-1-*N*-methyl-2-nitro-1*H*-imidazole (6)

Reduction of acid 5

To a solution of carboxylic acid **5** (1.46 g, 8.54 mmol) in anhydrous tetrahydrofuran (15 ml), was added triethylamine (1.87 ml, 13.5 mmol) and the mixture was cooled in a dry ice-acetone bath at –20 °C. Isobutyl chloroformate (1.77 ml, 13.7 mmol) was added dropwise and stirring was maintained for 2 h at –20 °C. Before addition of sodium borohydride (1.62 g, 42.9 mmol), the mixture was cooled in a bath of salt water at -20°C and water (15 ml) was slowly added over a period of 1 h 30 while maintaining temperature around -5 to -10 °C. After warm-up to 0 °C, precipitate was filtered and washed with tetrahydrofuran (10 ml). The filtrate was concentrated and the residue was purified by column chromatography (ethyl acetate) to give alcohol **6** (1 g, 6.36 mmol) as a yellow powder. Yield 75%; mp 140-142 °C (lit. 141-143 °C) [3]; Rf 0.50 (SiO_2, ethyl acetate); ^{1}H NMR (200 MHz, DMSO-d_6) δ 7.11 (s, 1H, CH), 5.50 (t, 1H, ^3J = 5.4 Hz, OH), 4.53 (d, 2H, ^3J = 5.4 Hz, C$\underline{H}_2$OH), 3.91 (s, 3H, NCH_3).

Reduction of ester 4

To a solution of ester **4** (2.18 g, 11.0 mmol) in a mixture of tetrahydrofuran and methanol (80/20, v/v, 26 ml) cooled at 0 °C was added dropwise a suspension containing sodium borohydride (1.25 g, 32.9 mmol) previously suspended in tetrahydrofuran (60 ml) and lithium bromide (2.88 g, 33.2 mmol) dissolved in water (12 ml) while maintaining the temperature around 0°C. The temperature was allowed to rise gradually to room temperature and the reaction mixture was stirred for 19 h, before addition of a saturated aqueous solution of ammonium chloride (10 ml). The mixture was extracted with ethyl acetate (3 × 150 ml) and the combined organic fractions were dried over magnesium sulphate, filtered. The solvent was evaporated under reduced pressure before purification by silica gel chromatography using ethyl acetate as eluent with a gradient of methanol of 0 to 2% to yield alcohol **6** (1.20, 76.2 mmol). Yield 70%.

O-[(1-Methyl-2-nitro-1*H*-imidazol-5-yl)methyl]-*N,N*-bis(2-chloroethyl)-*N*'-[3-dimethylamino)propyl]phosphorodiamidate (7)

To a solution of *N*-methyl-2-nitroimidazolyl methanol **6** (357 mg, 2.27 mmol) in freshly distilled anhydrous tetrahydrofuran (10 ml) was added lithium bis(trimethylsilyl)amide (1 M in tetrahydrofuran, 2.5 ml, 2.5 mmol) at −78 °C under an inert atmosphere. The reaction mixture was allowed to stir around 5 min, at −78 °C, and a solution of bis(2-chloroethyl)phosphoramidic dichloride (631 mg, 2.45 mmol) in tetrahydrofuran (7 ml) was added all at once at −78 °C. After 1 h of stirring at -78 °C (reaction progress monitored by ^{31}P NMR), *N,N*-dimethylamino-1-propylamine (486 µL, 3.85 mmol) was added and the stirring maintained at -78 °C for 1 h. The reaction solution was quenched with water (20 ml) and after return to room temperature, was extracted with ethyl acetate (3 × 20 ml). The organic extracts were combined, washed with brine (80 ml), dried over magnesium sulphate, filtered and concentrated under reduced pressure. Column chromatography of the crude product on silica gel (ethyl acetate/ethanol/ammonia, 50/50/1 to 50/50/5, v/v/v) afforded compound **7** (486 mg, 1.09 mmol) as a thick yellow oil. Yield 48%; Rf 0.40 (SiO_2, ethyl acetate/ethanol, 50/50, v/v with 12% ammonia); IR (ATR) ν 1492, 1353, 1221, 1013 cm^{-1}; ^{1}H NMR (500 MHz, CD_3OD) δ 7.24 (s, 1H, CH), 5.12 (d, 2H, ^3J = 8.1 Hz, OC<u>H</u>$_2$), 4.06 (s, 3H, NC<u>H</u>$_3$), 3.62-3.70 (m, 4H, NCH$_2$C<u>H</u>$_2$Cl), 3.34-3.48 (m, 4H, NC<u>H</u>$_2$CH$_2$Cl), 2.91 (td, 2H, $^3J_{H-P}$ = 10.9 Hz, ^3J = 6.9 Hz, C<u>H</u>$_2$NHP), 2,42 (m, 2H, C<u>H</u>$_2$N), 2,28 (s, 6H, N(C<u>H</u>$_3$)$_2$), 1.70 (qt, 2H, ^3J = 7.3 Hz, CH$_2$C<u>H</u>$_2$CH$_2$); ^{13}C NMR (50 MHz, CD_3OD) δ 135.0 (<u>C</u>$_{Ar}$CH$_2$), 129.1 (<u>C</u>H$_{Ar}$), 58.0 (N<u>C</u>H$_2$), 57.5 (d, $^2J_{C-P}$ = 4.6 Hz, O<u>C</u>H$_2$), 50.3 (d, $^2J_{C-P}$ = 4.8 Hz, N<u>C</u>H$_2$CH$_2$Cl), 45.3 (N(<u>C</u>H$_3$)$_2$), 43.1 (d, $^3J_{C-P}$ = 1.4 Hz, NCH$_2$<u>C</u>H$_2$Cl), 40.1 (<u>C</u>H$_2$NHP), 35.1 (N<u>C</u>H$_3$), 30.2 (d, $^3J_{C-P}$ = 5.8 Hz, CH$_2$<u>C</u>H$_2$CH$_2$); ^{31}P NMR (202 MHz, CD_3OD) δ 18.24; HRMS m/z 445.1271 [M + H]$^+$ (counted for [$C_{14}H_{28}Cl_2N_6O_4P$]$^+$ 445.1287).

O-[(1-Methyl-2-nitro-1*H*-imidazol-5-yl)methyl]-*N,N*-bis(2-chloroethyl)-*N'*-propylphosphorodiamidate (8)

To a solution of *N*-methyl-2-nitroimidazolyl methanol **6** (600 mg, 3.82 mmol) in freshly distilled anhydrous tetrahydrofuran (14 ml) was added lithium bis(trimethylsilyl)amide (1 M in tetrahydrofuran, 4.2 ml, 4.2 mmol) at −78 °C under an inert atmosphere. The reaction mixture was allowed to stir around 5 min, at −78 °C, and a solution of bis(2-chloroethyl)phosphoramidic dichloride (1.08 g, 4.17 mmol) in tetrahydrofuran (12 ml) was added all at once at −78 °C. After 1 h 40 of stirring at -78 °C, *n*-propylamine (618 µL, 7.52 mmol) was added and the stirring maintained at -78 °C for 20 min. The reaction solution was diluted with water (30 ml) and then extracted with ethyl acetate (3 × 30 ml). The organic extracts were combined, washed with brine (100 ml), dried over magnesium sulphate, filtered and concentrated under reduced pressure. The residue was purified by column chromatography on silica gel (ethyl acetate/ethanol, 98/2, v/v) to afford **8** (995 mg, 65%) as a thick yellow oil: Rf 0.40 (SiO_2, ethyl acetate/ethanol, 98/2, v/v); IR (ATR) ν 1489, 1350, 1218, 1004, 834 cm^{-1};^{1}H NMR (400 MHz, $CDCl_3$) δ 7.17 (s, 1H, CH), 5.00 (dd, 1H, $^3J_{H-P}$ = 7,1 Hz, ^2J = 13,3 Hz, OC<u>H</u>$_2$), 5.07 (dd, 1H, $^3J_{H-P}$ = 7.9 Hz, ^2J = 13.3 Hz, OC<u>H</u>$_2$), 4.05 (s, 3H, NC<u>H</u>$_3$), 3.59-3.62 (m, 4H, NCH$_2$C<u>H</u>$_2$Cl), 3.36-3.45 (m, 4H, NC<u>H</u>$_2$CH$_2$Cl), 2.79-2.88 (m, 2H, C<u>H</u>$_2$NHP), 2.70-2.79 (m, 1H, NHP), 1.50 (st, 2H, ^3J = 7.3 Hz, CH$_2$C<u>H</u>$_2$CH$_3$), 0.88 (t, 3H, ^3J = 7.4 Hz, CH$_2$CH$_2$<u>CH</u>$_3$); ^{13}C NMR (50 MHz, $CDCl_3$) δ 133.0 (d, $^3J_{C-P}$ = 7.8 Hz, <u>C</u>$_{Ar}$CH$_2$), 129.1 (CH$_{Ar}$), 56.1 (d, $^2J_{C-P}$ = 3.5 Hz, O<u>C</u>H$_2$), 49.2 (d, $^2J_{C-P}$ = 4.6 Hz, N<u>C</u>H$_2$CH$_2$Cl), 42.9, 42.7 (m, NCH$_2$<u>C</u>H$_2$Cl, <u>C</u>H$_2$NHP), 34.5 (N<u>C</u>H$_3$), 25.1 (d, $^3J_{C-P}$ = 6.5 Hz, CH$_2$<u>C</u>H$_2$CH$_2$), 11.3 (s, CH$_2$CH$_2$<u>C</u>H$_3$); ^{31}P NMR (202 MHz, $CDCl_3$) δ 18.24; HRMS m/z 402.0844 [M + H]$^+$ (counted for [$C_{12}H_{23}Cl_2N_5O_4P$]$^+$ 402.0865).

3-({[bis(2(chloroethyl)amino][(1-methyl-2-nitro-1*H*-imidazol-5-yl)methoxy]phosphoryl}amino)-*N,N,N*-trimethylpropane-1-aminium iodide (8-QA).

A mixture of *O*-[(1-methyl-2-nitro-1*H*-imidazol-5-yl)methyl]-*N,N*-bis(2-chloroethyl)-*N'*-[3-dimethylamino)propyl]phosphorodiamidate **7** (494 mg, 1.11 mmol) and methyl iodide (380 µL, 6.10 mmol) in freshly distilled anhydrous tetrahydrofuran (20 ml) was stirred in a sealed flask, under an inert atmosphere, for 3 h 30 at room temperature. After evaporation to dryness under reduced pressure, compound **8-QA** (646 mg, 99%) was obtained as a very hygroscopic yellow solid; Rf 0.03 (SiO_2, ethyl acetate/ethanol, 50/50, v/v with 12% ammonia); IR(ATR) ν 3441,1490, 1352, 1223, 1193, 1012 cm^{-1}; NMR ^{1}H (500 MHz, CD_3OD) δ 7.28 (s, 1H, C<u>H</u>), 5.20 (dd, 1H, $^3J_{H-P}$ = 8.5 Hz, ^2J = 13.4 Hz, OC<u>H</u>$_2$); 5.16 (dd, 1H, $^3J_{H-P}$ = 8.5 Hz, ^2J = 13.4 Hz, OC<u>H</u>$_2$), 4.08 (s, 3H, NC<u>H</u>$_3$), 3.65-3.73 (m, 4H, NCH$_2$C<u>H</u>$_2$Cl), 3.36-3.51 (m, 6H, NC<u>H</u>$_2$CH$_2$Cl, C<u>H</u>$_2$N$^+$), 3.18 (s, 9H, N(C<u>H</u>$_3$)$_3$), 3.01 (td, 2H, ^3J = 6.4 Hz,$^3J_{H-P}$ 11.9 Hz, C<u>H</u>$_2$NHP), 1.96-2.05 (m, 2H, CH$_2$C<u>H</u>$_2$CH$_2$); ^{1}H NMR (500 MHz, CD_3OD); ^{13}C NMR (50 MHz, CD_3OD) δ 134.9 (d, $^3J_{C-P}$ = 7.5 Hz, C$_{Ar}$CH$_2$O), 129.4 (CH$_{Ar}$), 65.8 (CH$_2$N$^+$), 58.0 (d, $^2J_{C-P}$ = 4.7 Hz, O<u>C</u>H$_2$), 54.0, 54.0, 53.9 ((CH$_3$)$_3$), 50.0 (d, $^2J_{C-P}$ = 4.7 Hz, N<u>C</u>H$_2$CH$_2$Cl), 43.3 (d, $^3J_{C-P}$ = 1.5 Hz, NCH$_2$<u>C</u>H$_2$Cl), 38.7 (<u>C</u>H$_2$NHP), 35.5 (N<u>C</u>H$_3$), 26.2 (d, $^3J_{C-P}$ = 5.1 Hz, CH$_2$<u>C</u>H$_2$CH$_2$); ^{31}P NMR (202 MHz, CD_3OD) δ 17.95; HRMS m/z 459.1429 [M]$^+$ (counted for [$C_{15}H_{30}Cl_2N_6O_4P$]$^+$ 459.1443).

O-benzyl-*N,N*-bis(2-chloroethyl)-*N'*-[3-(dimethylamino)propyl]phosphorodiamidate (9)

A solution of benzyl alcohol (1.5 ml, 14.4 mmol) in anhydrous toluene (4 ml) was added dropwise and under an inert atmosphere to a stirred ice-cooled suspension of sodium hydride (60 wt% in mineral oil, 577 mg, 14.4 mmol) in anhydrous toluene (10 ml). The reaction mixture was allowed to stir overnight in an ice-cold bath. The sodium benzylate suspension thus obtained was added dropwise to a solution of bis(2-chloroethyl) phosphoramidic dichloride **6** (3.73 g, 14.3 mmol) in ice-cooled anhydrous toluene (15 ml). After 4 hours of stirring, *N,N*-dimethylamino-1-propylamine (3.6 ml, 28.6 mmol) was added and the stirring maintained for 2 hours at 0°C. The reaction mixture was evaporated under reduced pressure and the residue was purified by column chromatography on silica gel (equivolume ethyl acetate/ethanol with 5% ammonia to yield compound **9** (5.08 g, 11.7 mmol) as a thick yellow oil. Yield 88%; Rf 0.08 (SiO_2, ethyl acetate/ethanol, 8/2, v/v); IR (ATR) ν 3153, 1672, 1390, 1216, 1007, 695 cm^{-1}; ^1H NMR (500 MHz, DMSO-d_6) δ 10.80 (se, 1H, N<u>H</u>$^+$), 7.32-7.52 (m, 5H, Ph), 5.11 (td, 1H, ^3J= 6.9 Hz, $^2J_{H-P}$ = 11.2 Hz, CH_2N<u>H</u>P), 4.91 (m, 2H, OC<u>H</u>$_2$Ph), 3.61-3.70 (m, 4H, NCH_2C<u>H</u>$_2$Cl), 3.25-3.35 (m, 4H, NC<u>H</u>$_2$$CH_2$Cl), 3.05 (m, C<u>H</u>$_2NH^+$), 2.83 (qd, 2H, ^3J= 6.6 Hz, $^3J_{H-P}$ = 11.4 Hz, C<u>H</u>$_2$NHP), 2.69 (s, 6H, (C<u>H</u>$_3$)$_2$), 1.82 (qt, 2H, ^3J= 6.9 Hz, CH_2C<u>H</u>$_2$$CH_2$); ^{13}C NMR (50 MHz, DMSO-d_6) δ 137.8 (d, $^3J_{C-P}$ = 7.4 Hz, <u>C</u>$_{Ar}$), 128.9, 128.5, 128.0 (<u>C</u>H$_{Ar}$), 66.6 (d, $^2J_{C-P}$ = 4.7 Hz, O<u>C</u>H$_2$Ph), 54.85 (<u>C</u>H$_2$NH$^+$), 49.0 (d, $^2J_{C-P}$ = 4.3 Hz, N<u>C</u>H$_2$$CH_2$Cl), 43.0 (d, $^3J_{C-P}$ = 0.9 Hz, NCH_2<u>C</u>H$_2$Cl), 42.5 ((<u>C</u>H$_3$)$_2$), 38.0 (<u>C</u>H$_2$NHP), 26.5 (d, $^3J_{C-P}$ = 5.3 Hz, CH_2<u>C</u>H$_2$$CH_2$); ^{31}P NMR (202 MHz, DMSO-d_6) δ 17.23; HRMS m/z 396.1369 $[M+H]^+$ (counted for $[C_{16}H_{29}Cl_2N_3O_2P]^+$ 396.1369).

3-({(Benzyloxy)[bis(2-chloroethyl)amino]phosphoryl}amino)-*N,N,N*-trimethylpropane-1-aminium iodide (10-QA)

To a solution of compound **9** (1 g, 2.31 mmol) in anhydrous tetrahydrofuran (23 ml) were added sodium carbonate (1.6 g, 15.1 mmol) and methyl iodide (778 μL, 12.6 mmol). The suspension was stirred in a sealed flask, under an inert atmosphere, for 2 h at room temperature. The reaction mixture was filtered and the precipitate washed with methanol (10 ml). The filtrate was concentrated under reduced pressure to yield compound **10-QA** (966 mg, 1.79 mmol) as a very hygroscopic white solid. Yield 78%; Rf 0.02 (SiO_2, ethanol/ammonia, 90/10); IR(ATR) ν 3189, 1663, 1382, 1217, 1017 cm^{-1}; ^1H NMR (500 MHz, DMSO-d_6) δ 7.32-7.43 (m, 5H, Ph), 5.08 (td, 1H, ^3J = 6.8 Hz, $^2J_{H-P}$ = 10.8 Hz, CH_2N<u>H</u>P), 4.91 (d, 2H, ^3J = 7.0 Hz, OC<u>H</u>$_2$Ph), 3.62-3.70 (m, 4H, NCH_2C<u>H</u>$_2$Cl), 3.24-3.36 (m, 6H, NC<u>H</u>$_2$$CH_2$Cl, C<u>H</u>$_2N^+$), 3.06 (s, 9H, (C<u>H</u>$_3$)$_3$), 2.83 (qd, 2H, ^3J = 6.4 Hz, $^2J_{H-P}$ = 11.4 Hz, C<u>H</u>$_2$NHP), 1.79-1.88 (m, 2H, CH_2C<u>H</u>$_2$$CH_2$); ^{13}C NMR (50 MHz, CD_3OD) δ 138.1 (d, $^3J_{C-P}$ = 7.3 Hz, <u>C</u>$_{Ar}$), 129.7, 129.4, 129.0 (<u>C</u>H$_{Ar}$), 68.6 (d, $^2J_{C-P}$ = 5.1 Hz, O<u>C</u>H$_2$Ph), 65.8 (<u>C</u>H$_2$N$^+$), 53.9, 53.8, 53.7 ((<u>C</u>H$_3$)$_3$), 50.3 (d, $^2J_{C-P}$ = 4.6 Hz, N<u>C</u>H$_2$$CH_2$Cl), 43.2 (d, $^3J_{C-P}$ = 1.3 Hz, NCH_2<u>C</u>H$_2$Cl), 38.7 (<u>C</u>H$_2$NHP), 26.2 (d, $^3J_{C-P}$ = 5.0 Hz, CH_2<u>C</u>H$_2$$CH_2$); ^{31}P NMR (202 MHz, DMSO-d_6) δ 17.12; HRMS m/z 410.1532 $[M]^+$ (counted for $[C_{17}H_{31}Cl_2N_3O_2P]^+$ 410.1525).

REFERENCES

1. Johnston TP, Mccaleb GS. Carcinogenic Nitrosamines: Hundred-Gram Preparations of *N*-Nitrosodiethanolamine and α-Ureidodimethylnitrosamine. Synthesis. 1984; 1984: 311–3. https://doi.org/10.1055/s-1984-30821.
2. Hay MP, Wilson WR, Denny WA. Design, Synthesis and Evaluation of Imidazolylmethyl Carbamate Prodrugs of Alkylating Agents. Tetrahedron. 2000; 56: 645–57. https://doi.org/10.1016/S0040-4020(99)01031-5.
3. O'Connor LJ, Cazares-Körner C, Saha J, Evans CNG, Stratford MRL, Hammond EM, Conway SJ. Design, synthesis and evaluation of molecularly targeted hypoxia-activated prodrugs. Nat Protoc. 2016; 11: 781–94. https://doi.org/10.1038/nprot.2016.034.
4. O'Connor LJ, Cazares-Körner C, Saha J, Evans CNG, Stratford MRL, Hammond EM, Conway SJ. Efficient synthesis of 2-nitroimidazole derivatives and the bioreductive clinical candidate Evofosfamide (TH-302). Org Chem. 2015; 2: 1026–9. https://doi.org/10.1039/C5QO00211G.
5. Cavalleri B, Ballotta R, Lancini GC. Synthesis of 1-alkyl-2-nitroimidazole-5-carboxaldehydes. J Heterocycl Chem. 1972; 9: 979–84. https://doi.org/10.1002/jhet.5570090502.

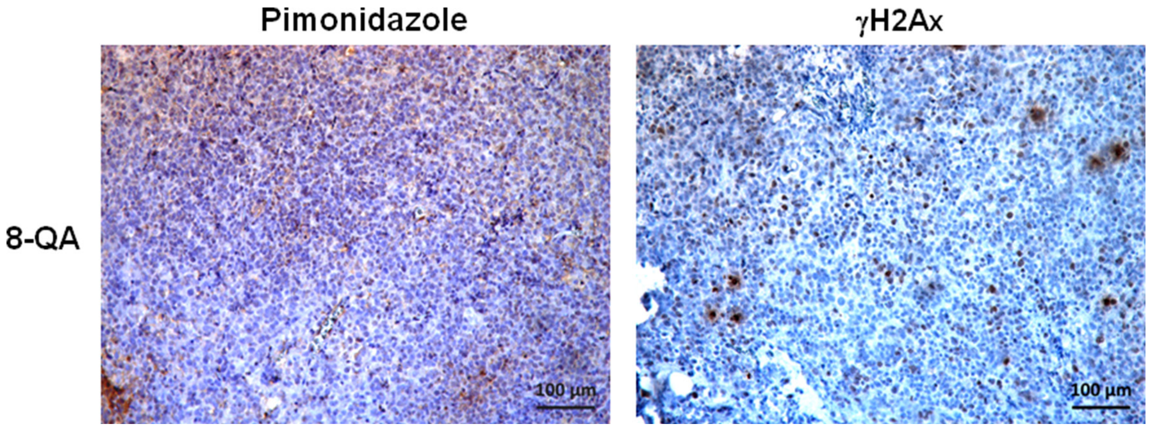

Supplementary Figure 1: Distribution of DNA damages in normoxic areas of 8-QA treated tumors. In normoxic areas (pimoniazole negative), a weak γH2Ax staining was observed.

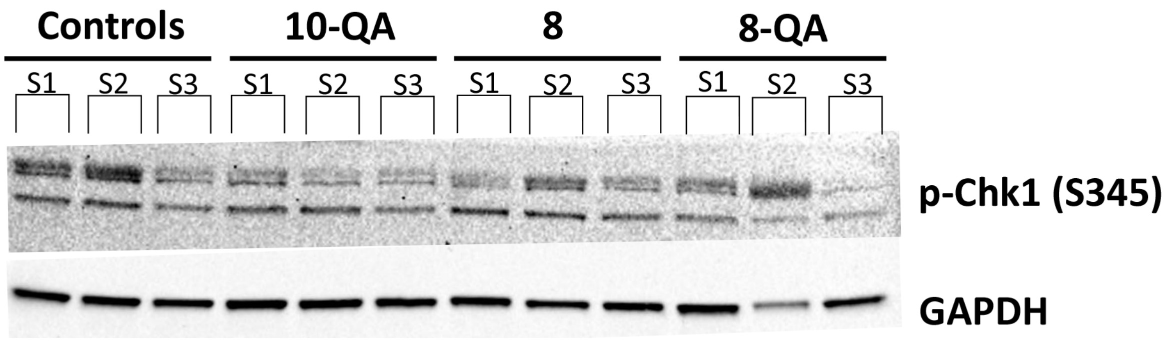

Supplementary Figure 2: Western blot analyses of p-Chk1 (S345) on tumors sampled one day after last injection. No increased of p-CHK1 (S345) was observed in **8-QA** treated samples, suggesting that the increased of γH2Ax observed by immunostaining could be attributed to **8-QA** alkylating drug. Individual bands (S1, S2, S3) correspond to 3 independant tumor samples per group of treatment.
